# Multiple imputation of missing data under missing at random: compatible imputation models are not sufficient to avoid bias if they are mis-specified

**DOI:** 10.1016/j.jclinepi.2023.06.011

**Published:** 2023-06-19

**Authors:** Elinor Curnow, James R. Carpenter, Jon E. Heron, Rosie P. Cornish, Stefan Rach, Vanessa Didelez, Malte Langeheine, Kate Tilling

**Affiliations:** a Department of Population Health Sciences, Bristol Medical School, University of Bristol, Bristol, UK; b Medical Research Council Integrative Epidemiology Unit at the University of Bristol, University of Bristol, Bristol, UK; c Department of Medical Statistics, London School of Hygiene and Tropical Medicine, University of London, London, UK; d Medical Research Council Clinical Trials Unit at University College London, University of London, London, UK; e Department of Epidemiological Methods and Etiological Research, Leibniz Institute for Prevention Research and Epidemiology - BIPS, Bremen, Germany; f Faculty of Mathematics/Computer Science, University of Bremen, Bremen, Germany; g Department of Biometry and Data Management, Leibniz Institute for Prevention Research and Epidemiology - BIPS, Bremen, Germany; h Department of Health and Consumer Protection, Senator for Health, Women and Consumer Protection, Bremen, Germany

**Keywords:** Missing data, Multiple imputation, Complete records analysis, Compatibility, Mis-specification, Predictive mean matching

## Abstract

**Objectives:**

Epidemiological studies often have missing data, which are commonly handled by multiple imputation (MI). Standard (default) MI procedures use simple linear covariate functions in the imputation model. We examine the bias that may be caused by acceptance of this default option and evaluate methods to identify problematic imputation models, providing practical guidance for researchers.

**Study Design and Setting:**

Using simulation and real data analysis, we investigated how imputation model mis-specification affected MI performance, comparing results with complete records analysis (CRA). We considered scenarios in which imputation model mis-specification occurred because (i) the analysis model was mis-specified or (ii) the relationship between exposure and confounder was mis-specified.

**Results:**

Mis-specification of the relationship between outcome and exposure, or between exposure and confounder, can cause biased CRA and MI estimates (in addition to any bias in the full-data estimate due to analysis model mis-specification). MI by predictive mean matching can mitigate model mis-specification. Methods for examining model mis-specification were effective in identifying mis-specified relationships.

**Conclusion:**

When using MI methods that assume data are MAR, compatibility between the analysis and imputation models is necessary, but not sufficient to avoid bias. We propose a step-by-step procedure for identifying and correcting mis-specification of imputation models.

## 1 Introduction

Missing data are ubiquitous in health and social research. While there are a number of methods for analyzing partially observed datasets (including inverse probability weighting, IPW, and maximum likelihood, ML, methods such as the expectation-maximization algorithm), multiple imputation (MI) is the most flexible, general, and commonly used [1]. When imputation models are appropriately specified, MI gives valid inferences if data are missing completely at random (MCAR) or missing at random (MAR), but not (unless additional information is provided by the analyst) if data are missing not at random (MNAR) (Table 1). Appropriate specification of the imputation model for each partially observed variable means that (a) it must be compatible with the analysis model (i.e., it must contain the same variables in the same form, including any interaction terms implied by the analysis model) [3–6], and (b) it must be a correctly specified model for the variable being imputed. Guidelines [1,7] have tended to focus on compatibility rather than correct specification.

Here, through a comprehensive set of simulation studies and a reanalysis of the trial data, we investigate the likely bias in MI estimates due to imputation model mis-specification. Here, “bias” refers to the difference between the MI estimate and the full data estimate i.e., we aim to evaluate specifically how much bias occurs due to imputation model mis-specification, *in addition* to any bias due to using a mis-specified analysis model. As part of this, we evaluate methods (which make use of the complete records) for identifying imputation model mis-specification. We conclude with an easy-to-follow, step-by-step procedure for identifying and correcting any imputation model mis-specification when performing MI.

## 2 Motivating example

We were motivated by the analysis of a randomized controlled trial assessing the effect of a “use acupuncture” treatment policy for chronic headache [8,9]. The primary outcome (headache score at 1 year follow-up) was missing for 25% of participants. The primary and sensitivity analyses (using complete records analysis, CRA, and MI, respectively) assumed linear relationships between the primary outcome and the continuous covariates (baseline headache score, age, and chronicity). However, an exploratory analysis described in the original publication suggested a nonlinear relationship between baseline headache score and the primary outcome, which was not accounted for in the CRA or MI analyses. Since the imputation model contained the same variables as the analysis model, in the same form, we say that the imputation model was compatible with the analysis model. However, since the relationship between baseline headache score and the primary outcome appears to be nonlinear, we say that the imputation model was mis-specified.

## 3 Simulation study

### 3.1 Aims

We used simulation to assess the performance of MI when the imputation model was mis-specified, comparing the results with CRA. The aims of our study were (a) to quantify the bias of, and accuracy of inference for, the exposure coefficient (*β_X_*) and (b) to quantify the sensitivity and utility of methods for examining imputation model mis-specification when either the chosen analysis model, or the relationship between the exposure and confounder, was mis-specified with respect to the data generating model. We defined the true value of *β_X_* as the full data estimate using all simulated datasets combined. Hence, bias is defined as the difference between the CRA or MI estimate and the full data estimate.

### 3.2 Analysis model

In our analysis models, we assumed a linear relationship between an outcome *Y* (or *logit*{P(*Y* = 1)} when *Y* was binary—note that for brevity, we will simply refer to *Y* hereafter), a single exposure *X*, and a single confounder *C*, i.e., we fitted the model *E*(*Y*) = *β*_0_ + *β*_X_ *X* + *β_C_ C* when *Y* was continuous, and *logit*{P(*Y* = 1)} = *β*_0_ + *β_X_ X* + *β_C_ C* when *Y* was binary.

In the presence of missing data (for either *Y, X*, or *C*), we compared three strategies.

(i) CRA.
(ii) MI with an imputation step as follows: missing values are replaced by draws from a linear or logistic regression model, for continuous or binary variables respectively. This is the default method when using *mi impute* in Stata [10] or *proc mi* in SAS [11], although note that predictive mean matching is the default method for continuous variables when using *mice* in R [12].
(iii) MI by “type 1” predictive mean matching [13] (hereafter referred to as PMM). This method was used for continuous variables only.

Following current guidelines [7], we performed MI and PMM compatible with the analysis model, i.e., we assumed linear relationships between *Y*, *X*, and *C* in the imputation model(s). We used a donor pool of size five for PMM and performed 30 imputations for both MI and PMM.

### 3.3 Data generation

We generated data with a nonlinear relationship between *Y* and *X* (scenarios 1 and 4, with *X* continuous, *C* binary, and *Y* continuous or binary), between *Y* and *C* (scenario 2, all variables continuous), and between *X* and *C* (scenario 3, all variables continuous). In scenario 1, we varied the strength of the nonlinear relationship between *Y* and *X*. In all other scenarios, we used fixed values for nonlinear relationships (Table 2). We used 1,000 simulations in each scenario, and each simulated dataset contained 1,000 subjects. The standard deviation of the per-simulation estimates of the exposure coefficient, ${\hat{\beta }}_{X},$ was at most 0.2 (see Supplementary Material, Tables S3—S6). Hence, 1,000 simulations gave a Monte Carlo (MC) standard error [14] of the estimated bias of ${\hat{\beta }}_{X}$ of at most 0.006.

In each scenario, we considered two separate settings (each with a single partially observed variable) e.g., in scenario 1, missingness of either *Y* or *C* was caused by *X*. In each setting, missingness did not depend on the outcome *Y*, given the observed data (hence the CRA estimate was, in principle, unbiased) and data were MAR (hence the MI estimate was, in principle, unbiased). See Supplementary Material Section S1 for further explanation. We considered four different strengths of the missingness association. The proportion of missing data was approximately 30% in each scenario. We generally did not vary the proportion of missing data because it is already well known that any bias in the estimated association between *X* and *Y* will increase with the proportion of missing data [15]. However, to illustrate this effect, we repeated the analysis with 10% and 50% missing data in scenario 1 (see Table 2).

### 3.4 Methods for examining model mis-specification

In each scenario, we explored possible mis-specification of both the analysis and imputation models by applying either a linear or logistic regression model mis-specification method, as appropriate, to the complete records. For example, in scenario 1 (continuous *Y*, binary *C*), we (i) examined the analysis model specification using a mis-specification method for a linear regression model, and (ii) examined the imputation model for *C* using a mis-specification method for a logistic regression model. Note that, after examining the specification of the analysis model, it was not necessary to additionally examine imputation model mis-specification when *Y* was partially observed. This was because relationships between *Y, X*, and *C* were the same in the analysis and imputation models in our simulations. In other applications, therefore, these methods would only additionally be needed for the imputation model when it included auxiliary variables (Table 1).

In each scenario, we used the following methods for examining model mis-specification:

1. Linear regression model mis-specification: we examined the association between residuals and the best-fitting fractional polynomial (FP) of the fitted values.
2. Logistic regression model mis-specification: we used Pregibon’s “link” method [16].

We identified these methods as the best performing of nine available methods for examining model mis-specification (six for linear regression and three for logistic regression) (see Supplementary Material Section S2 for further details).

All analyses were conducted using Stata (17.0, Stata-Corp LLC, College Station, TX). Stata code to perform the simulation study is included in Supplementary Material, Section S5.

## 4 Simulation study results

### 4.1 Results for scenario 1: estimating the exposure coefficient

**Fig. 1:**
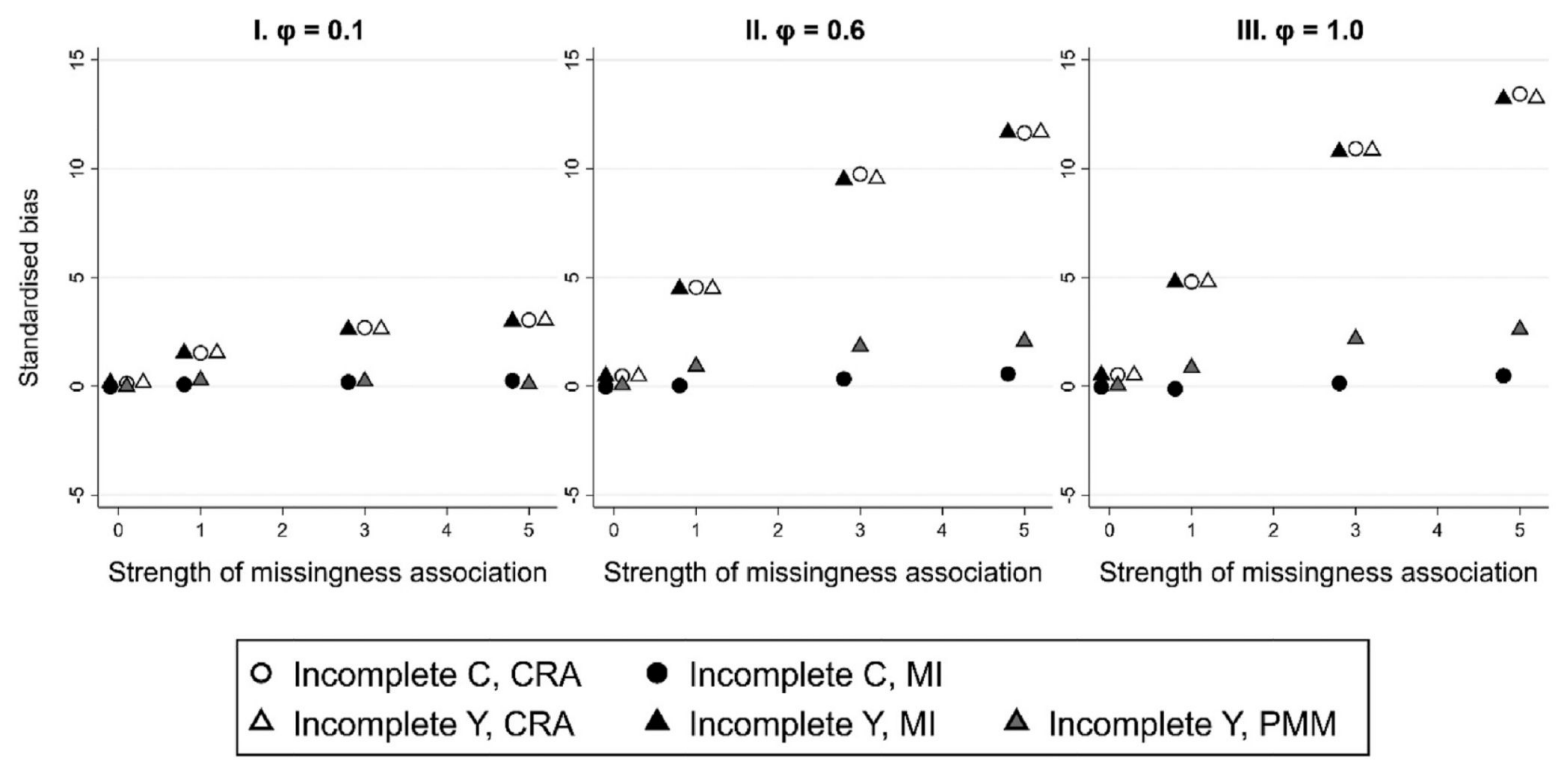
Standardized bias of complete records analysis (CRA), multiple imputation (MI), and predictive mean matching (PMM) estimates of parameter *β_X_*, plotted against the strength of the missingness association, for different strengths of the nonlinear association (ϕ) between X and Y, given a quadratic relationship between continuous variables Y and X, and either C or Y partially observed. Some overlapping points have been horizontally jittered. Monte Carlo SE of bias is at most 0.006.

Results for scenario 1 (quadratic relationship between continuous variables *Y* and *X*) are summarized in Figure 1. We show results using the three analysis approaches (CRA, MI and PMM) when the analysis model (incorrectly) assumes linear relationships between *Y*, *X*, and *C*, and missingness of *C* or *Y* depends on (fully observed) exposure *X*. We present standardized bias of ${\hat{\beta }}_{X},$ defined as $\text{bias/SD(}{\hat{\beta }}_{X}\text{)}$ (for brevity, hereafter referred to as “bias”). Full results showing standardized bias, bias of ${\hat{\beta }}_{X},\;SD({\hat{\beta }}_{X}),$ and model-based standard error are included in Supplementary Material Table S3. We use CRA or MI with a subscript “Y”, “X,” or “C” to denote which variable was partially observed for a particular estimate e.g., CRA_Y_ refers to the CRA estimate when *Y* is partially observed.

In scenario 1, the chosen analysis model does not specify the relationship between *X* and *Y* correctly. The imputation model for *Y* is also mis-specified because it has the same form as the analysis model. Therefore, as expected, CRA_Y_, CRA_C_, and MI_Y_ estimates are biased (see Supplementary Material Section S3 for further explanation of this result). As expected, bias increased with the proportion of missing data (see Supplementary Material Figure S5). Figure 1 shows that bias is of similar magnitude for CRA_Y_, CRA_C_, and MI_Y_ estimates, and increases with the strength of the nonlinear association (*ϕ*) (increasing across plots I-III), as well as with the strength of the missingness association (*τ*) (shown on the x-axis in each plot). Consistent with previous studies [13,17], PMM_Y_ estimates are less biased than MI_Y_ when the imputation model is mis-specified, although some bias remains in PMM_Y_ estimates unless the non-linear association is weak. Since, in general, each imputation model should include all relationships implied by the correct analysis model, in the same form as in the correct analysis model, the imputation model for *C* is also mis-specified (here, this is because a quadratic association is induced between *C* and *X* by conditioning on *Y*). However, the bias of the MI_C_ estimates is small because the induced nonlinear association between *C* and *X* is fairly weak, even when other associations (between pairs of variables and with missingness) are strong (e.g., when *ϕ* = 1.0 and *τ* = 5.0, the parameter estimate for the squared term is −0.085, 95% CI: −0.093, −0.077).

### 4.2 Results for scenarios 2—4: estimating the exposure coefficient

**Fig. 2:**
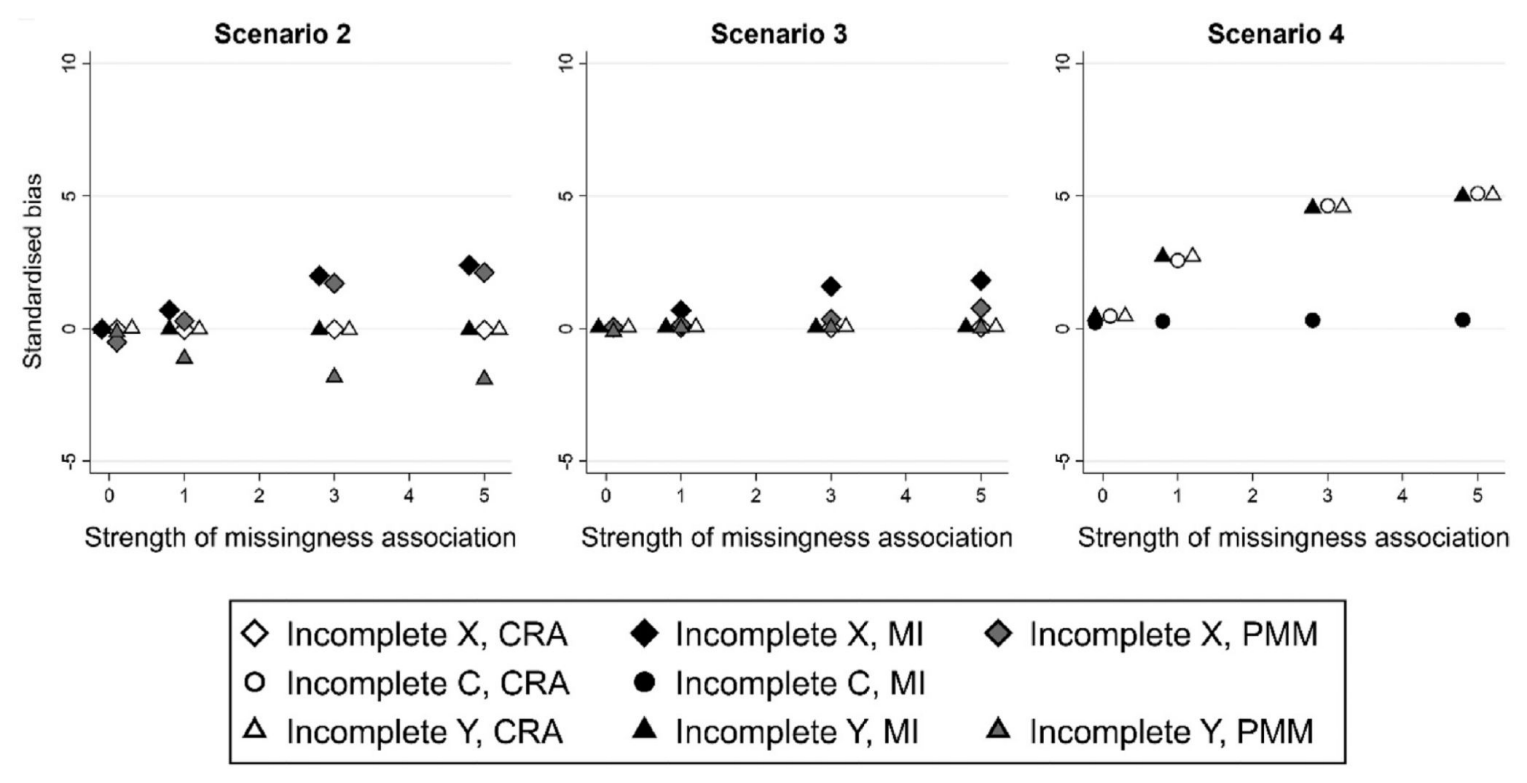
Standardized bias of complete records analysis (CRA), multiple imputation (MI), and predictive mean matching (PMM) estimates of parameter *β_X_*, plotted against the strength of the missingness association, given (i) Scenario 2: quadratic relationship between continuous variables Y and C, (ii) Scenario 3: quadratic relationship between continuous variables X and C, and (iii) Scenario 4: quadratic relationship between binary Y and continuous X, and either X, C, or Y partially observed. Some overlapping points have been horizontally jittered. Monte Carlo SE of bias is at most 0.006.

Figure 2 illustrates results for scenarios 2—4 (respectively: quadratic relationship between continuous variables *Y* and *C* and missingness of *X* or *Y* depends on *C*; quadratic relationship between continuous variables *X* and *C* and missingness of *X* or *Y* depends on *C*; quadratic relationship between binary *Y* and continuous *X* and missingness of *C* or *Y* depends on *X*). As before, we present standardised bias of ${\hat{\beta }}_{X},$ plotted against the strength of the missingness association (*τ*) (see Supplementary Material, Tables S4—S6, for full results).

As in scenario 1, Figure 2 shows that any bias in *β_X_* estimates increases in magnitude as *τ* increases. In scenario 2, CRA_Y_, CRA_X_, and MI_Y_ estimates of *β_X_* have little or no bias because the relationship between *Y* and *X* is correctly specified (even though the relationship between *Y* and *C* is mis-specified). When *X* is partially observed, the imputation model for *X* is mis-specified. Similar to scenario 1, mis-specification occurs because a quadratic relationship is induced between *X* and *C* by conditioning on *Y*. However, in contrast to scenario 1, the induced non-linear association between *X* and *C* is fairly strong (despite using similar effect sizes in data generation to those in scenario 1 — see Table 2), e.g., when *τ* = 5.0, the parameter estimate for the squared term is -0.208, 95% CI: -0.211, -0.205. Hence, MI_X_ estimates have some bias. As in scenario 1, PMM estimates have some bias, with more bias than the equivalent MI estimate when the relationship with *X* is correctly specified in the imputation model (PMM_Y_ vs. MI_Y_) and less bias when the relationship with *X* is mis-specified (PMM_X_ vs. MI_X_).

In scenario 3, the relationships between *Y, X*, and *C* are correctly specified in the analysis model, and in the imputation model for *Y*. Hence, CRA_Y_, CRA_X_, and MI_Y_ estimates of *β_X_* are unbiased as expected. PMM_Y_ and PMM_X_ estimates also have little bias. MI_X_ estimates have some bias, because the relationship between *X* and *C* is mis-specified in the imputation model for *X*. In scenario 4 (which has the same set-up as scenario 1, except that *Y* is binary rather than continuous), results are very similar to scenario 1.

### 4.3 Results: methods for examining model mis-specification

For each scenario, Table 3 shows sensitivity and type 1 error when using Methods 1 and 2 (see Section 3.4) to examine model mis-specification. We define sensitivity and type 1 error, respectively, as the proportion of *P* values for each method < 0.05 when the relevant model was mis-specified (i.e., when it incorrectly assumed linear relationships between *Y*, *X*, and *C*) and the proportion of *P* values for each method < 0.05 when the model was correct (i.e., when it additionally included any squared terms implied by the correct analysis model). Results are shown for a single value of the strength of the missingness association (*τ* = 1.0), when examining the analysis model with *Y* partially observed (continuous in scenarios 1—3, and binary in scenario 4) and when examining the imputation model with continuous *X* or binary *C* partially observed. Results were similar for other values of *τ* (see Supplementary Material Tables S7—S9 for full results).

Both methods were sensitive to model mis-specification when the nonlinear association was strong (e.g., Scenario 1, *ϕ* = 1.0). However, the logistic regression model mis-specification method was less sensitive when the nonlinear association was weak (e.g., *C* partially observed in scenario 1, *ϕ* = 0.1). Reassuringly, when the analysis or imputation model was correctly specified, type 1 error was ≤0.05 for each method.

## 5 Analysis of the motivating example

To illustrate our methods, we used data from the randomized controlled trial described earlier [8,9]. Here, our focus was on the relationship between headache score at baseline (*hs0*) and headache score at 1 year follow-up (*hs1*).

### 5.1 Methods

We performed a linear regression of *hs1* on *hs0*, adjusting for treatment allocation group (being randomized to receive acupuncture plus standard care, vs. receiving standard care alone), and other baseline variables: age, sex, diagnosis (migraine or tension-type headache), and chronicity (number of years of headache disorder). Following the main trial analysis design, we included continuous variables (*hs0*, age, and chronicity) as linear terms in the analysis model.

The outcome, *hs1*, was observed for 301 (75%) of the 401 trial participants. Baseline data were completely observed. We used MI to handle missing values of *hs1*. Initially, we used an imputation model that was compatible with, but no richer than, the analysis model i.e., we used an imputation model identical to the analysis model.

We then assessed possible bias in the *hs0* coefficient estimate due to imputation model mis-specification, firstly by applying the linear regression model mis-specification method described earlier, and secondly by comparing CRA, MI and PMM estimates. To correct any imputation model mis-specification, we used FP selection to identify the best functional form for each continuous variable in the imputation model. We then updated the MI estimate after including any required nonlinear terms (for consistency with the main trial analysis design, we updated the imputation model but not the analysis model). For MI and PMM, we used 25 imputations, reflecting the percentage of participants with a missing outcome [18]. For PMM, we used a donor pool of size five. As per the simulation study, only one variable was partially observed and so no iterations were performed in the imputation procedures. Stata code to perform the real data analysis is included in Supplementary Material Section S6.

Note that the MI methods considered here are only valid if the outcome is not MNAR. However, an MNAR mechanism is plausible in the context of this trial e.g., participants who experienced less severe headaches may have been less motivated to continue to participate in the trial. This issue was explored in the original trial–all participants were contacted at 1 year and all but 24 provided a global (one-off) estimate of headache severity, which was used in a sensitivity analysis. More recently, Cro et al. [19] performed extensive sensitivity analyses using the same data. For simplicity and only for illustration, here we assume the outcome is MAR, given the observed baseline data.

### 5.2 Results

Table 4 shows the estimated mean increase in *hs1* (conditional on all other baseline variables) per unit increase in *hs0*, using the different analysis approaches. When analysis and imputation models included only linear terms, CRA and MI estimates were very similar. However, the PMM estimate was slightly larger. Exploration of the model specification suggested that the analysis model (and consequently the imputation model) was mis-specified (*P* = 0.001).

Applying FP selection for the exposure and all other continuous baseline variables in turn suggested a quadratic relationship between *hs0* and *hs1* (parameter estimate for the squared term was 0.007, 95% CI: 0.004, 0.011), but little evidence of a nonlinear association between age (*P* = 0.530) or chronicity (*P* = 0.409) and *hs1*. After including a linear and a squared term for *hs0* in the imputation model for *hs1* (but leaving the analysis model unchanged), the updated MI estimate was slightly larger than the CRA and original MI estimates, and closer to the PMM estimate. There was no longer evidence of model mis-specification (*P* = 0.915). These results are consistent with findings from scenario 1 of our simulation study, namely, that mis-specification of the relationship between exposure and outcome gives biased CRA and MI estimates. However, in this particular setting, the nonlinear association between exposure and outcome was weak and hence the bias in the CRA and MI estimates was small.

## 6 Discussion

In this paper, we have used a comprehensive simulation study to show that CRA and MI estimates can be biased in situations in which many researchers would expect these approaches to be valid, namely when data are MAR, the analysis and imputation models are compatible, and missingness does not depend on the outcome variable [20].

Our results showed that CRA and MI estimates of the exposure coefficient can be substantially biased if the relationship between the exposure and outcome is mis-specified, or the relationship between the exposure and confounder is mis-specified in the imputation model for the exposure. This is because the (mis-specified) relationship with the exposure in records with missing data differs from the (mis-specified) relationship in records with fully observed data (see Supplementary Material Section S3). Hence, the full data value of the exposure coefficient cannot be recovered from the partially observed data. In our simulations, we found that bias was much smaller if the relationship between the outcome and confounder was mis-specified. However, bias may have been larger in more complex settings e.g., if there was an exposure-confounder interaction. In general, we found that the magnitude of bias increased with the strength of the nonlinear relationship. As in previous studies [13,17], we found that PMM estimates were less biased than MI estimates when the imputation model was mis-specified.

Further, we found that methods for examining model mis-specification, applied to the complete records, were effective in identifying mis-specified relationships. It is valid to use the complete records to check for analysis and imputation model mis-specification (assuming positivity), given data are MAR and missingness does not depend on the analysis outcome. If the model is incorrect, the complete records provide evidence to reject it, provided the number of complete records is large enough (i.e., given sufficient power), relative to the severity of mis-specification. When the analysis outcome is partially observed, the analysis model and the imputation model are the same (in the absence of auxiliary data). Hence, after exploring mis-specification of the analysis model, it is not necessary to additionally explore mis-specification of the imputation model for the outcome. When a covariate is partially observed, provided that its missingness does not depend on the covariate itself conditional on the variables in its imputation model, it is also valid to perform model checks for the imputation model using the complete records. In settings similar to those in our simulations, we recommend a residual-based method (using FP selection) to identify model mis-specification in a linear regression model, and Pregibon’s “link” method to identify model mis-specification in a logistic regression model, although (as in other settings), any other appropriate method could be applied.

We used a testing procedure to assess the performance of methods for examining model mis-specification in our simulation study. However, in general, we do not advocate the use of a *P* value “test” for model mis-specification. Where there may be evidence of model mis-specification (a small *P* value, or a wide confidence interval around a nonlinear term), we recommend conducting a sensitivity analysis using a different model specification. Where the power to detect mis-specification is lower (e.g., with small sample size), it may be useful to use a more conservative *P* value threshold to guide the decision on changing the model specification. Furthermore, applying a method for examining model mis-specification must be followed by thorough data exploration to identify, as far as possible, the correct model, rather than attempting to mitigate for model mis-specification e.g., by using PMM, or by fitting a spline function for each continuous predictor in the imputation model. This is because MI using a correctly specified model will yield more precise estimates than PMM or an over-specified model [21]. We recommend using statistical methods for model mis-specification, rather than visual inspection alone (although this can provide insight into the nature of the mis-specification), because model mis-specification may not be visually apparent from the observed data.

In addition to correcting any imputation model mis-specification, a further decision is whether to change the analysis model in light of mis-specification. There may be valid clinical and scientific reasons for retaining linear relationships between all variables in the analysis model (such as pre-specification in a clinical trial setting, or particular interest in the average marginal effect). Any decision to change the analysis model must take into account the study aims, the strength and complexity of the true relationships between variables, the strength of the missingness association, and which variables are partially observed. Our work shows that such decisions are better informed when the imputation model is correctly specified.

A strength of our approach is that we have considered a range of scenarios in which model mis-specification is likely to occur in real data, varying the strengths of both the nonlinear and missingness associations. A limitation of our study is that in each of our scenarios, only one relationship is mis-specified and only a single variable has missing values. Assessing model mis-specification when multiple relationships are mis-specified and/or multiple variables have missing values is likely to be a more complex process. In this case, it is important to check for imputation model mis-specification for each incomplete variable in turn. A further limitation of our study (as with any simulation study) is that we have only considered mis-specification of the functional form for each variable in the analysis/imputation model. We have not considered other possible types of model mis-specification, such as not including interactions, mis-specification of the link function in the analysis model, or mis-specification due to using models that are more complex than the true model. However, we would expect our findings to extend to these situations. Finally, in this study, we have focused on MI due to its flexibility and common use in practice. Other available methods, such as ML, IPW, or the use of “doubly robust” estimators (which combine MI with IPW) do not rely on correct specification of an imputation model. However, such methods instead require correct specification of the marginal or weighting model and generally yield less precise estimates than MI when this uses auxiliary information and a correctly specified (and not overspecified) imputation model [21].

We conclude that when using MI methods that assume MAR, compatibility between the analysis and imputation models is necessary, but is not sufficient to avoid bias. It is important to check (as far as possible) that each imputation model is correctly specified, bearing in mind that an incorrect imputation model can be a consequence of an incorrect analysis model. Table 5 outlines a possible approach, suggested by our results, that can be used to identify and correct any imputation model mis-specification when performing MI.

## Supplementary Material

Supplementary Material

## Figures and Tables

**Table 1:** Missing data definitions

Term	Definition
Complete Records Analysis (CRA)	Analysis is restricted to subjects who have complete data for all variables in the analysis model.
Missing Completely At Random (MCAR)	The probability that data are missing is independent of the observed and missing values of variables in the analysis model, and of any related variables. Data can be MCAR if missingness is caused by a variable independent of those in the analysis model e.g., if missingness is for administrative reasons.
Missing At Random (MAR)	Given the observed data, the probability that data are missing is independent of the true values of the incomplete variable. Any systematic differences between the observed and missing values can be explained by associations with the observed data.
Missing Not At Random (MNAR)	If data are not MCAR nor MAR, data are said to be MNAR. The probability that data are missing depends on the (unobserved) values of the incomplete variable, even after conditioning on the observed data.
Multiple Imputation (MI)	MI is a method for handling missing data. It consists of three steps: An imputation model is fitted to the observed data (this is usually some form of regression model). The missing values are replaced with draws (“imputed”) from its predictive distribution (after first perturbing the model parameters). This imputation stage is carried out multiple (M) times, to give M completed datasets. The analysis model is fitted to each of the M completed datasets. The M sets of results are combined using Rubin’s rules [2], to correctly account for the uncertainty about the missing values.
Predictive Mean Matching (PMM)	PMM is an MI approach that uses an alternative method in step 1 of the MI process: instead of imputing missing values directly from the conditional predictive distribution of the missing data given the observed data, each missing value is replaced with an observed value randomly chosen from a donor pool anchored on the conditional predicted mean.
Auxiliary variable	A variable that is not in the analysis model but that is included as a predictor in the imputation model to recover information about the missing data.

**Table 2:** Data generating mechanism (DGM) and missingness mechanism used in the simulation study in scenarios 1—4

Seen.	DGM for *Y*	DGM for *X*	DGM for *C*	Partially observed variables^a^	Missingness meehanism^b^
1	*Y* is continuous and depends on *X, X*^2^ and *C*: *Y* = *Y* = -0.4 + 0.4 *X* + 0.8 *C* + *ϕ X*^2^ + *ε_Y_* where *ϕ* = 0.1, 0.6 or 1.0	*X* is continuous and depends on *C*: *X* = *C* + *ε_X_*	*C* is binary, with probability 0.5 of a value of 0 or 1	*C* or *Y*	Missingness depends on *X*: *logit*{P(*R_Δ_* = 1)} = *τ* (*α* + *X*)
2	*Y* is continuous and depends on *X*, *C* and *C*^2^: *Y* = -0.4 + 0.4 *X* + 0.8 *C* + 0.6 *C*^2^ + *ε_Y_*	*X* is continuous and depends on *C*: *X* = *C* + *ε_X_*	*C* is normally distributed, with mean 0.5, variance 1	*X* or *Y*	Missingness depends on *C: logit*{P(_R_Δ__ = 1)} = *τ* (*α* + *C*)
3	*Y* is continuous and depends on *X* and *C*: *Y* = -0.4 + 0.4 *X* + 0.8 *C* + *ε_Y_*	*X* is continuous and depends on *C*^2^: *X* = *C*^2^ + *ε_X_*	*C* is normally distributed, with mean 0.5, variance 1	*X* or *Y*	Missingness depends on *C: logit*{P(*R_Δ_* = 1)} = *τ* (*α* + *C*)
4	*Y* is binary and depends on *X, X*^2^ and *C*: *logit*{P(*Y* = 1)} = -0.4 + 0.4 *X* + 0.8 *C* + 0.5 *X*^2^	*X* is continuous and depends on *C*: *X* = *C* + *ε_X_*	*C* is binary, with probability 0.5 of a value of 0 or 1	*C* or *Y*	Missingness depends on *X: logit*{P(*R_Δ_* = 1)} = *τ* (*α* + *X*)

*Abbreviation: logit*, logistic function. In each scenario, we assumed the error terms *ε_Y_* and *ε_X_* were uncorrelated, with standard normal distributions (mean 0, variance 1). For each missingness mechanism, *α* was chosen empirically to give approximately 70% observed values (and additionally 50% and 90% observed values in scenario 1 when ϕ = 1.0), for each strength of missingness association (*τ*), *τ* = 0.1, 1, 3 or 5.

a Two separate situations were considered in each scenario: (i) the partially observed variable was directly involved in the mis-specified relationship and (ii) the partially observed variable was not directly involved. Values were set to missing for one variable only in each situation.

b P(*R_Δ_* = 1) denotes the probability that a value (of the partially observed variable) is observed, with *Δ* = *X, C* or *Y*.

**Table 3:** Sensitivity and type 1 error of methods for examining analysis model mis-specification when Y (continuous in scenarios 1—3, and binary in scenario 4) is partially observed, and imputation model mis-specification when continuous X or binary C are partially observed (method for continuous variables: fitting a degree two fractional polynomial in the regression of the residuals on the fitted values; method for binary variables: Pregibon’s “link” method)

Seenario	Partially observed variable	Sensitivity (type 1 error)
1, *ϕ* = 0.1	Y (continuous)	0.71 (0.01)
	C (binary)	0.10 (0.03)
1, *ϕ* = 0.6	Y (continuous)	1.00 (0.03)
	C (binary)	0.81 (0.02)
1, *ϕ* = 1.0	Y (continuous)	1.00 (0.03)
	C (binary)	0.96 (0.02)
2	Y (continuous)	1.00 (0.01)
	X (continuous)	1.00 (0.01)
3	Y^a^ (continuous)	NA (0.02)
	X (continuous)	1.00 (0.01)
4	Y (binary)	1.00 (0.04)
	C (binary)	0.04 (0.00)

Results for scenario 1 are shown for different strengths of the nonlinear association (ϕ) between X and Y. The strength of the missingness association (*τ*) = 1.0.

a In scenario 3, the chosen analysis model correctly specifies the *Y-X* and *Y-C* relationships.

**Table 4:** Mean increase in headache score at 1 year, per unit increase in baseline headache score, using different analysis approaches

Analysis approach	Mean increase in headache score at 1 yr, per unit increase in baseline headache score^a^	
	Estimate	95% CI
CRA	0.71	0.63–0.79
MI including linear terms only	0.70	0.62–0.79
PMM	0.74	0.66–0.83
After imputation model refinement:		
MI including linear and squared term for baseline headache score	0.73	0.65–0.81

a Adjusted for treatment group, age, sex, diagnosis, and chronicity.

**Table 5:** Procedure for identifying and correcting imputation model mis-specification when using MI

Step	Method
1	Examine the specification of the analysis model: Fit the analysis model to the complete records (perform a CRA) Use a method for assessing model mis-specification (e.g., regress the residuals on an FP of the fitted values if using a linear regression model, or Pregibon’s “link” method if using a logistic regression model)
2	If there is evidence of analysis model mis-specification AND any of the partially observed variables are continuous, compare CRA, MI and PMM estimates using the current specification of the analysis model. PMM estimates similar to CRA and MI estimates would suggest analysis model mis-specification has little impact on the estimate of interest.
	Note that this step should not be applied if model mis-specification is not identified in step 1 (as in this case, differences between the estimates may be due to bias in the PMM estimate).
3	Identify (as far as possible) the correct specification of the analysis model (e.g., by using FP selection for each continuous covariate in turn).
4	Decide whether to respecify the analysis model, or just imputation models, in light of analysis model mis-specification
5	For each partially observed variable in turn, examine the specification of the imputation model, ensuring that each imputation model is compatible with the corrected analysis model i.e., including variables in the same form, and including any interactions implied by the corrected analysis model.
6	Correct any imputation model mis-specification (e.g., using FP selection for each continuous predictor in turn in the fully conditional specification [22] imputation algorithm) — ensuring that each conditional model remains consistent with the others.
	Note that steps 5 and 6 do not need to be applied if only the outcome is partially observed and there are no auxiliary variables.
7	Perform MI using the corrected imputation models (and possibly, a corrected analysis model).

Procedure assumes that a linear or logistic regression model is fitted, that at least one analysis model covariate/imputation model predictor is continuous, that data are MAR, and that CRA is, in principle, valid.

## Data Availability

Stata code to generate and analyze the data as per the simulation study is included in Supplementary Material, Section S5. Stata code to analyze the real data example is included in Supplementary Material, Section S6. The real data are available at: https://www.ncbi.nlm.nih.gov/pmc/articles/PMC1489946/#S1.

## What is new?

### Key findings

- When using MI methods that assume data are MAR, compatibility between the analysis and imputation models is necessary, but is not sufficient to avoid bias.

### What this adds to what was known?

- Mis-specification of the relationship between outcome and exposure, or between exposure and confounder in the imputation model for the exposure, can cause substantial bias in the exposure coefficient estimate.
- Methods for examining model mis-specification can be used to identify mis-specified relationships in any setting in which CRA and MI assuming MAR are valid in principle.

### What is the implication and what should change now?

- Uncritical acceptance of simple linear covariate functions in the imputation model should be avoided.
- It is important to check (as far as possible) that each imputation model is correctly specified, bearing in mind that an incorrect imputation model can be a consequence of an incorrect analysis model.
