# Supplementary material for: Multiple imputation of missing data under missing at random: compatible imputation models are not sufficient to avoid bias if they are mis-specified
Source: J Clin Epidemiol. Author manuscript; Available in PMC 2024 Jan 4. (PMC7615471; doi:10.1016/j.jclinepi.2023.06.011)
Supplement: Supplementary Material [file EMS192889-supplement-Supplementary_Material.docx]

**Multiple imputation of missing data under missing at random: compatible imputation models are not sufficient to avoid bias if they are mis-specified**

**Authors:** Elinor Curnow, James R Carpenter, Jon E Heron, Rosie P Cornish, Stefan Rach, Vanessa Didelez, Malte Langeheine, Kate Tilling

**Supplementary Material**

***Section S1: Validity of model checking using complete records***

In our simulation scenarios, if the analysis and imputation models were correctly specified, CRA estimates would be unbiased (regardless of the sample size) because missingness does not depend on the outcome *Y*, given the observed data. This can be verified by inspecting the “missingness” directed acyclic graph (DAG) [1] for each scenario (Figure S1).

*Figure S1. For scenarios 1 to 4 in the simulation study, directed acyclic graphs depicting the relationship between Y, X, C, and missingness indicator R_Δ_ (Δ = Y or C in Scenarios 1 and 4; Δ = Y or X in Scenarios 2 and 3) in CRA.*

*Lines indicate related variables, with arrows indicating the direction of the relationship; absent lines represent conditional independencies. Boxes indicate variables conditioned on in CRA.*

*
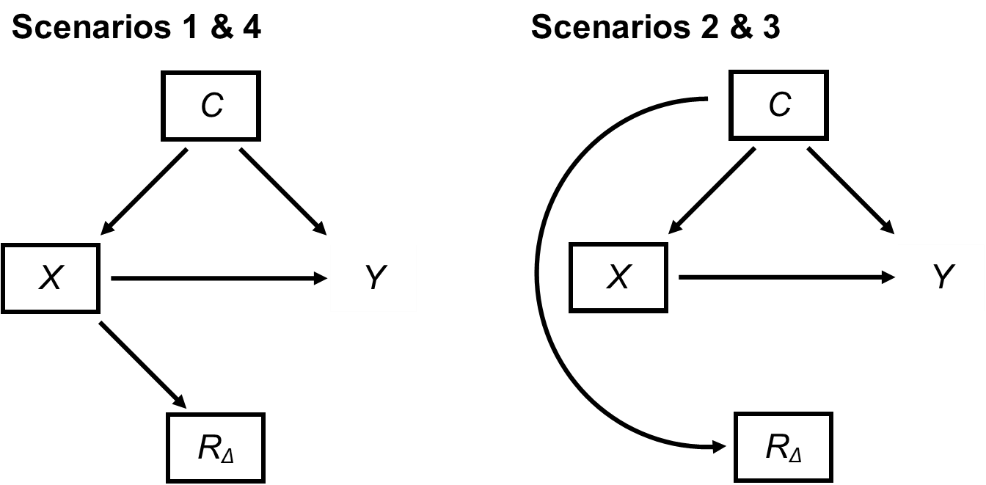
*

In each DAG in Figure S1, solid lines indicate the relationships between *Y*, *X*, *C*, and missingness indicator *R_Δ_* (*Δ = Y* or *C* in Scenarios 1 and 4; *Δ = Y* or *X* in Scenarios 2 and 3), with arrows indicating the direction of the relationship. Boxes indicate the variables conditioned on in CRA. Boxes around *X* and *C* indicate that these variables are regressors in the analysis model. The box around *R_Δ_* indicates that we only include complete records in CRA.

In all scenarios, missingness does not depend on *Y* (*i.e.* there is no open path between *Y* and *R_Δ_*), given *X* and *C*. Hence, CRA is valid (though not necessarily efficient). In particular, this means that it is valid to use the complete records to check for analysis model mis-specification (assuming positivity). If the model is incorrect, the complete records provide evidence to reject it, provided the number of complete records is large enough (*i.e.* given sufficient power), relative to the severity of mis-specification.

Similarly, if the analysis and imputation models were correctly specified, MI estimates would be unbiased because data are MAR. When *Y* is partially observed, the analysis model and the imputation model are the same (in the absence of auxiliary data), and hence it is not necessary to additionally examine mis-specification of the imputation model for *Y*. When *C* or *X* are partially observed (in Scenarios 1 & 4, and 2 & 3, respectively), both *C* and *X* are independent of *R_Δ_*, given the variables in their respective imputation models. Again, this could be verified by inspecting missingness DAGs for the imputation models for *C* or *X* (note that these would contain the same variables and relationships as in Figure S1, but the variables conditioned on would differ: we would condition on *X* and *Y* in the imputation model for *C*, and on *C* and *Y* in the imputation model for *X*. To avoid repetition, these DAGs are not shown). Thus, it is also valid to perform model checks for the imputation model for *C* or *X* using the complete records.

Note that in analysis of real data, there may be additional, unmeasured variables related to *Y*, *X*, *C*, and their missingness (in our simulation studies, we have assumed that all required variables are at least partially observed). There may also be several partially observed variables and/or missingness may depend on more than one variable. In this case, checks for analysis and imputation model mis-specification will be more complex, but we can still use missingness DAGs to establish whether it is valid to check for model mis-specification using the complete records. If the missingness DAG indicates that this is not valid (*e.g.* because *Y* is MNAR), we cannot use complete records to identify model mis-specification (and in any case, changing the functional form of variables in the analysis and/or imputation models will not mitigate for data MNAR).

Further note that if all covariates in the analysis model, or all predictors in the imputation model, are binary, then the form of mis-specification we consider here cannot occur *e.g.* if the outcome is continuous and partially observed, and all covariates in the analysis model are binary, bias due to mis-specification of the functional form of the covariates in the analysis model cannot occur (assuming a saturated model *i.e.* that there are no missing higher order interaction terms). However, in this scenario, if the binary exposure is partially observed, then mis-specification of the relationship between the exposure and continuous outcome in the imputation model could result in bias, and should be checked.

***Section S2. Assessing the performance of methods for examining model mis-specification***

*Methods*

In Scenario 1, we considered nine methods for examining model mis-specification (six for linear regression and three for logistic regression), as follows:

*Linear regression model mis-specification*

We applied the following methods, designed to assess the validity of the linear regression model:

1. Pregibon [2] “link” method for examining whether the correct link function has been used. This was originally designed to examine the specification of the outcome. However, this method can also be used to indicate mis-specification of the covariate model.
2. Shapiro-Wilk [3] method for assessing the normality of residuals.
3. Breusch–Pagan/Cook–Weisberg [4, 5] (heteroskedasticity) method for assessing constant variance of residuals.
4. Fractional polynomial (FP) degree-two method for assessing the association between residuals and the best-fitting FP of the fitted values. The best-fitting FP is identified using FP selection [6], choosing from a set of polynomial functions based on the model deviance. In degree two FP selection, a pair of polynomials is fitted (choosing the best-fitting pair from the set of all possible pairwise combinations). We chose the fractional powers from the set {–2, –1, –0.5, 0, 0.5, 1, 2, 3, where a power of zero is the log function}.
5. FP degree-one method for assessing the association between residuals and fitted values. This is a variant of Method 4, fitting a single polynomial instead of a pair of polynomials.
6. The grouped residuals method fits a regression of the model residuals on the grouped fitted values (grouped according to the quintiles of the distribution of the fitted values).

*Logistic regression model mis-specification*

1. Pregibon [2] “link” method for examining whether the correct link function has been used. This method can be used for logistic as well as linear regression models.
2. Hinkley’s [7] method. This involves comparing the original fitted model to a model including a FP of the logit of the predicted probabilities from the original fitted model (*i.e.* a FP of the linear predictor).
3. Hosmer-Lemeshow [8] method. This method compares observed and expected event rates in grouped data, grouped according to quantiles of the predicted probabilities. We used ten groups.

*Results*

For each method that we considered in Scenario 1, Tables S1 and S2 show (for linear and logistic regression models, respectively) the proportion of p-values < 0.05 when the relevant model was mis-specified (*i.e.* when it assumed linear relationships between *Y*, *X*, and *C*)*,* and the proportion of p-values < 0.05 when the model was correct (*i.e.* when it additionally included *X^2^*). We refer to these proportions as “sensitivity” and “type 1 error”, respectively. Results are shown when examining the analysis model with *Y* partially observed (Table S1) and when examining the imputation model for *C* with *C* partially observed (Table S2), for a single value of the strength of the missingness association (*τ* = 1), and three different strengths of non-linear association between *X* and *Y* (*φ* = 0.1, 0.6 or 1.0). Results were similar for other values of *τ* and when examining the analysis model with *C* partially observed (see Tables S7 and S8 for full results).

When the non-linear association was weak (*φ* = 0.1), in which case the bias of ${\hat{\text{β}}}_{\text{X}}$ across the analysis approaches was relatively small, most methods had low sensitivity to model mis-specification. However, of the methods for linear regression models, the link method was highly sensitive even when the model was only slightly mis-specified *i.e.* it highlighted mis-specification when the bias was not practically important. Therefore, in Scenarios 2-4 we used the FP degree-two method rather than the link method for linear regression models (because its sensitivity was more proportional to the bias of ${\hat{\text{β}}}_{\text{X}}$). When the non-linear association was stronger (*φ* = 0.6 or 1.0), all methods for linear regression models, except the heteroskedasticity method, detected model mis-specification in all simulations (Table S1, sensitivity = 1.00 in each case). Methods for logistic regression models (Table S2) were less sensitive in these situations, with the link method most sensitive to model mis-specification. Hence, we used the link method for logistic regression models in Scenarios 2-4. Reassuringly, when the analysis or imputation model was correctly specified, type 1 error was ≤ 0.05 for each method.

*Table S1. Sensitivity and type 1 error of various methods for examining linear regression model mis-specification. Results shown for Scenario 1 when examining the analysis model for different strengths of the non-linear association (φ) between X and Y, when the strength of the missingness association (τ) = 1 and Y was partially observed.*

| ***φ*** | **Sensitivity (type 1 error)** | | | | | |
| --- | --- | --- | --- | --- | --- | --- |
|  |  |  |  | **Model for regression of residuals on fitted values** | | |
|  | 1. **Link** | 1. **Shapiro-Wilk** | 1. **Hetero-skedasticity** | 1. **FP deg. 2 method** | 1. **FP deg. 1 method** | 1. **Grouped regression** |
| 0.1 | 0.92 (0.00) | 0.04 (0.05) | 0.05 (0.04) | 0.71 (0.01) | 0.09 (0.04) | 0.33 (0.02) |
| 0.6 | 1.00 (0.01) | 1.00 (0.05) | 0.35 (0.04) | 1.00 (0.03) | 1.00 (0.04) | 1.00 (0.02) |
| 1.0 | 1.00 (0.03) | 1.00 (0.04) | 0.51 (0.05) | 1.00 (0.03) | 1.00 (0.04) | 1.00 (0.02) |

FP, fractional polynomial

*Table S2. Sensitivity and type 1 error of various methods for examining logistic regression model mis-specification. Results shown for Scenario 1 when examining the imputation model for C for different strengths of the non-linear association (φ) between X and Y, when the strength of the missingness association (τ) = 1 and C was partially observed.*

| ***φ*** | **Sensitivity (type 1 error)** | | |
| --- | --- | --- | --- |
|  | 1. **Link** | 1. **Hinkley*** | 1. **Hosmer-Lemeshow** |
| 0.1 | 0.10 (0.03) | 0.03 (0.03) | 0.05 (0.04) |
| 0.6 | 0.81 (0.02) | 0.46 (0.02) | 0.45 (0.04) |
| 1.0 | 0.96 (0.02) | 0.87 (0.01) | 0.73 (0.04) |

* The model for the Hinkley method did not converge in 3% of simulations

***Section S3. Explanation of bias in CRA and MI estimates in simulation study scenarios***

In Figures S2-S4, we use plots of the simulated data to provide an intuitive explanation for why CRA and/or MI estimates of $\text{β}_{\text{X}}$ are biased in some of our simulation scenarios. Each plot uses data from 200 randomly selected simulated records. In each plot, whether a record has missing data or not is indicated by hollow or filled circles, respectively. Figure S2 shows plots of *Y* against *X* for Scenario 1, for different strengths of the non-linear association (*φ*) between *Y* and *X*, and for different strengths of the association (*τ*) between missingness (of *Y* or *C*) and *X*. The regression line for the regression of *Y* on *X*, assuming a linear relationship, is also shown for the full data analysis and for CRA.

*Figure S2. Plots of Y against X for Scenario 1, for different strengths of the non-linear association (φ) between Y and X, and for different strengths of the association (τ) between missingness and X. Regression lines, assuming a linear relationship between Y and X, are shown for the full data and for records with complete data.*

*Each plot uses data from 200 randomly selected simulated records.*


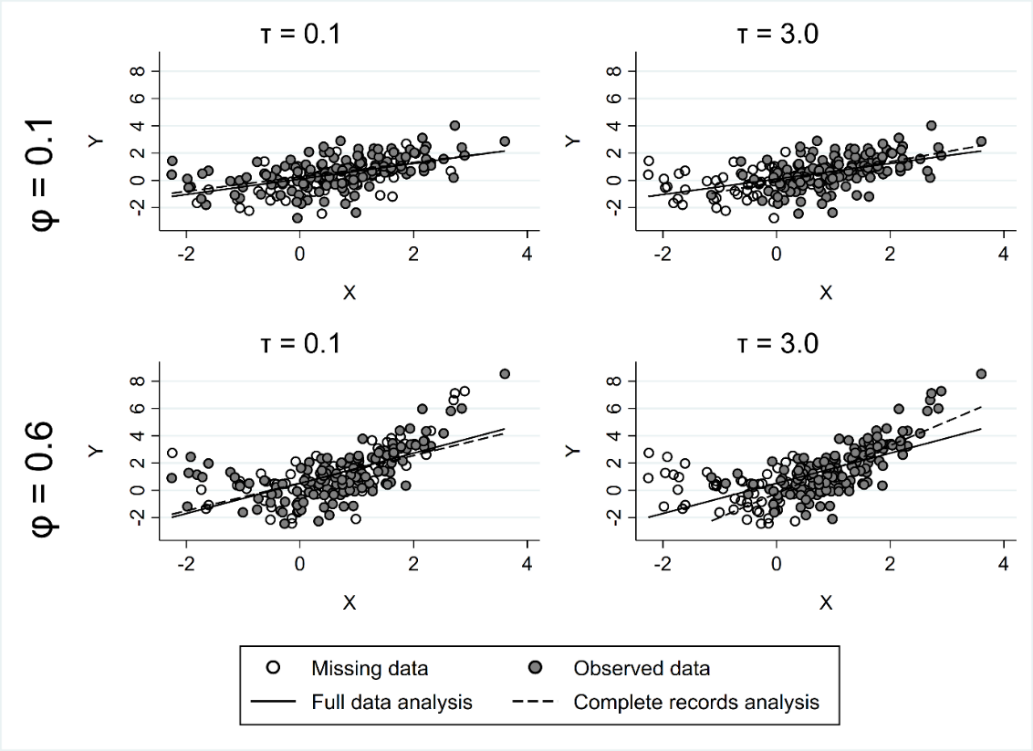


From the left-hand plots in Figure S2, we can see that when data are weakly MAR (when *τ =* 0.1), the CRA exposure coefficient (*i.e.* the slope) is similar to the full data coefficient, even when the non-linear association is fairly strong (when *φ =* 0.6). However, when data are strongly MAR (when *τ =* 3.0) and the non-linear association is fairly strong (when *φ =* 0.6), records with missing data have a different linear *X-Y* relationship from records with fully observed data. Hence, the true (full data) value of the coefficient cannot be recovered from the observed data, neither in CRA, nor in MI. Note this is not the case when a quadratic (the correctly specified relationship) is fitted (Figure S3). The same argument can be used to explain the bias in both CRA and MI estimates in Scenario 4, in which there is also a non-linear relationship between *Y* and X, but *Y* is binary rather than continuous.

*Figure S3. Plot of Y against X for Scenario 1, when the non-linear association (φ) between Y and X = 0.6, and the strength of the association (τ) between missingness and X = 3.0. Regression lines, assuming a quadratic relationship between Y and* *X, are shown both for the full data and for records with complete data.*

*The plot uses data from 200 randomly selected simulated records.*


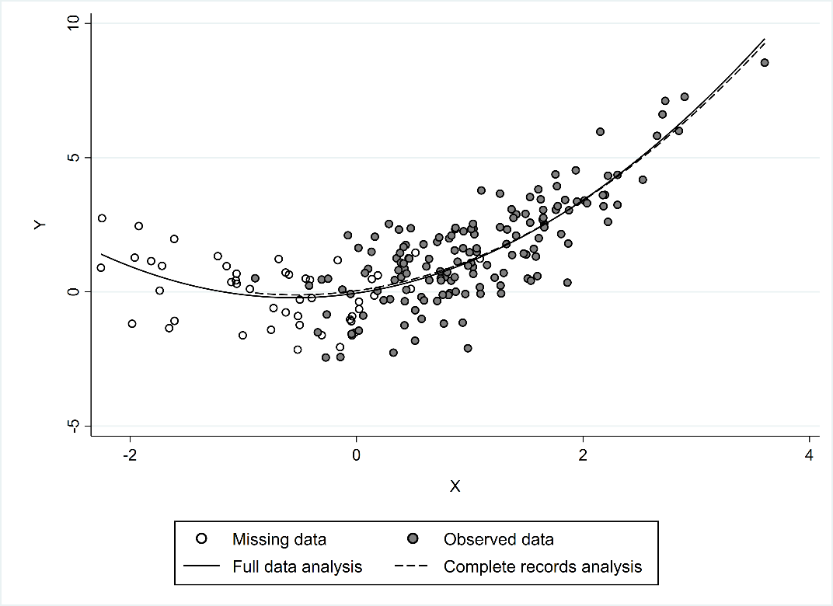


Conversely, Figure S4 (overleaf) shows plots of *Y* against *X* for Scenarios 2 and 3, when data are strongly MAR (when *τ* = 3.0). We can see that the CRA regression coefficient (*i.e.* the slope) is similar to the full data coefficient because the relationship between *X* and *Y* is linear in these scenarios. Hence, the true (full data) value of the exposure coefficient can be recovered from the observed data in both CRA, and MI when *Y* is partially observed (although not in MI when *X* is partially observed, because the relationship between *X* and *C* is mis-specified in the imputation model for *X*).

*Figure S4. Plots of Y against X for Scenarios 2 and 3,* *when the strength of the association (τ) between missingness and C = 3.0. Regression lines, assuming a linear relationship between Y and X, are shown both for the full data and for records with complete data.*

*Each plot uses data from 200 randomly selected simulated records*


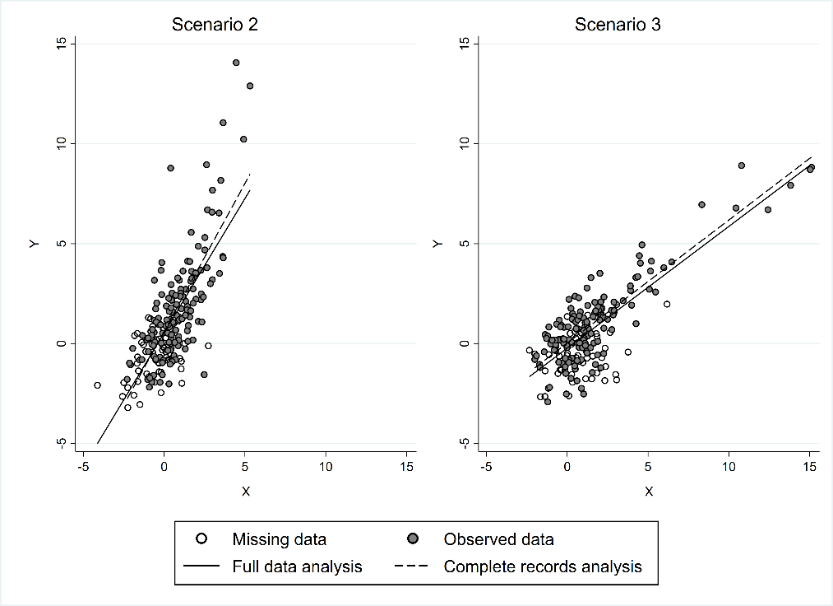


***References for Sections S1-S3***

[1] R. M. Daniel, M. G. Kenward, S. N. Cousens, and B. L. D. Stavola, "Using causal diagrams to guide analysis in missing data problems," *Stat. Methods Med. Res.,* vol. 21, no. 3, pp. 243-256, 2012.

[2] D. Pregibon, "Data analytic methods for generalized linear models," PhD, University of Toronto, 1979.

[3] S. S. Shapiro and M. B. Wilk, "An analysis of variance test for normality (complete samples)," *Biometrika,* vol. 52, no. 3-4, pp. 591–611, 1965.

[4] T. S. Breusch and A. R. Pagan, "A simple test for heteroscedasticity and random coefficient variation," *Econometrica* vol. 47, pp. 1287-1294, 1979.

[5] R. D. Cook and S. Weisberg, "Diagnostics for heteroscedasticity in regression," *Biometrika,* vol. 70, pp. 1-10, 1983.

[6] P. Royston and D. G. Altman, "Regression using Fractional Polynomials of Continuous Covariates: Parsimonious Parametric Modelling," *Applied Statistics,* vol. 43, no. 3, pp. 429-467, 1994.

[7] D. Hinkley, "Transformation diagnostics for linear models," *Biometrika,* vol. 72, no. 3, pp. 487-96, 1985.

[8] D. W. Hosmer and S. Lemeshow, "Goodness of fit tests for the multiple logistic regression model," *Communications in Statistics,* vol. 9, no. 10, pp. 1043-1069, 1980.

***Section S4: Simulation study results***

*Table S3. Simulation results for Scenario 1: Y and X are continuous, C is binary, Y depends on C, X, and X^2^, and X depends on C.*

*Estimates of standardised bias (bias/ SD(*${\hat{\text{β}}}_{\text{i}}$*)), bias of* ${\hat{\text{β}}}_{\text{i}}$*, SD(*${\hat{\text{β}}}_{\text{i}}$*), and model-based standard error (SE) are shown (i = 1 or 2), for complete records analysis (CRA), MI using draws from a linear/logistic imputation model (MI), and MI using type 1 predictive mean matching (PMM).*

*Results are shown for different strengths of the association (τ) between X and missingness of C or Y, and for different strengths of the non-linear association (φ) between X and Y.*

*True values of the exposure and confounder coefficients for the fitted analysis model (E(Y) = β_0_ + β_1_ X + β_2_ C) are β_1_ = 0.4 + φ, β_2_ = 0.8.*

| ***φ*** | **Partially observed**  **variable** | ***τ*** | **Par.** | **Standardised Bias** | | | **Bias of** ${\hat{\text{β}}}_{\text{i}}$ | | | **SD(**${\hat{\text{β}}}_{\text{i}}$**)** | | | **Model-based SE** | | |
| --- | --- | --- | --- | --- | --- | --- | --- | --- | --- | --- | --- | --- | --- | --- | --- |
|  |  |  |  | **CRA** | **MI** | **PMM*** | **CRA** | **MI** | **PMM*** | **CRA** | **MI** | **PMM*** | **CRA** | **MI** | **PMM*** |
| 0.1 | C | 0.1 | *β_1_* | 0.130 | -0.015 | n/a | 0.005 | -0.001 | n/a | 0.042 | 0.036 | n/a | 0.039 | 0.034 | n/a |
|  |  | 0.1 | *β_2_* | -0.013 | -0.010 | n/a | -0.001 | -0.001 | n/a | 0.085 | 0.084 | n/a | 0.086 | 0.083 | n/a |
|  |  | 1.0 | *β_1_* | 1.534 | 0.089 | n/a | 0.065 | 0.003 | n/a | 0.043 | 0.035 | n/a | 0.041 | 0.034 | n/a |
|  |  | 1.0 | *β_2_* | -0.137 | 0.003 | n/a | -0.012 | 0.000 | n/a | 0.085 | 0.083 | n/a | 0.084 | 0.082 | n/a |
|  |  | 3.0 | *β_1_* | 2.699 | 0.191 | n/a | 0.130 | 0.007 | n/a | 0.048 | 0.035 | n/a | 0.047 | 0.034 | n/a |
|  |  | 3.0 | *β_2_* | -0.146 | 0.054 | n/a | -0.012 | 0.004 | n/a | 0.083 | 0.081 | n/a | 0.083 | 0.081 | n/a |
|  |  | 5.0 | *β_1_* | 3.047 | 0.255 | n/a | 0.154 | 0.009 | n/a | 0.051 | 0.035 | n/a | 0.050 | 0.034 | n/a |
|  |  | 5.0 | *β_2_* | -0.136 | 0.051 | n/a | -0.011 | 0.004 | n/a | 0.085 | 0.083 | n/a | 0.083 | 0.081 | n/a |
| 0.1 | Y | 0.1 | *β_1_* | 0.168 | 0.163 | -0.004 | 0.007 | 0.006 | 0.000 | 0.040 | 0.040 | 0.040 | 0.039 | 0.039 | 0.038 |
|  |  | 0.1 | *β_2_* | -0.030 | -0.027 | -0.053 | -0.003 | -0.002 | -0.005 | 0.083 | 0.084 | 0.085 | 0.086 | 0.087 | 0.086 |
|  |  | 1.0 | *β_1_* | 1.540 | 1.535 | 0.281 | 0.064 | 0.064 | 0.013 | 0.042 | 0.042 | 0.046 | 0.041 | 0.041 | 0.038 |
|  |  | 1.0 | *β_2_* | -0.101 | -0.104 | -0.333 | -0.008 | -0.009 | -0.029 | 0.084 | 0.085 | 0.087 | 0.084 | 0.085 | 0.084 |
|  |  | 3.0 | *β_1_* | 2.636 | 2.618 | 0.226 | 0.128 | 0.128 | 0.016 | 0.049 | 0.049 | 0.073 | 0.047 | 0.047 | 0.040 |
|  |  | 3.0 | *β_2_* | -0.147 | -0.148 | -0.684 | -0.012 | -0.012 | -0.060 | 0.082 | 0.082 | 0.088 | 0.083 | 0.083 | 0.083 |
|  |  | 5.0 | *β_1_* | 3.038 | 2.995 | 0.113 | 0.154 | 0.154 | 0.010 | 0.051 | 0.051 | 0.087 | 0.050 | 0.050 | 0.040 |
|  |  | 5.0 | *β_2_* | -0.150 | -0.152 | -0.825 | -0.012 | -0.013 | -0.082 | 0.083 | 0.084 | 0.099 | 0.083 | 0.084 | 0.084 |
| 0.6 | C | 0.1 | *β_1_* | 0.473 | -0.016 | n/a | 0.043 | -0.001 | n/a | 0.092 | 0.079 | n/a | 0.054 | 0.047 | n/a |
|  |  | 0.1 | *β_2_* | -0.126 | 0.020 | n/a | -0.014 | 0.002 | n/a | 0.111 | 0.109 | n/a | 0.122 | 0.117 | n/a |
|  |  | 1.0 | *β_1_* | 4.552 | 0.037 | n/a | 0.398 | 0.003 | n/a | 0.087 | 0.077 | n/a | 0.054 | 0.045 | n/a |
|  |  | 1.0 | *β_2_* | -0.618 | 0.795 | n/a | -0.067 | 0.099 | n/a | 0.109 | 0.124 | n/a | 0.111 | 0.120 | n/a |
|  |  | 3.0 | *β_1_* | 9.756 | 0.335 | n/a | 0.780 | 0.024 | n/a | 0.080 | 0.072 | n/a | 0.055 | 0.044 | n/a |
|  |  | 3.0 | *β_2_* | -0.791 | 2.008 | n/a | -0.076 | 0.248 | n/a | 0.096 | 0.124 | n/a | 0.097 | 0.121 | n/a |
|  |  | 5.0 | *β_1_* | 11.645 | 0.572 | n/a | 0.934 | 0.042 | n/a | 0.080 | 0.073 | n/a | 0.056 | 0.044 | n/a |
| 0.6 | C | 5.0 | *β_2_* | -0.775 | 2.452 | n/a | -0.072 | 0.296 | n/a | 0.093 | 0.121 | n/a | 0.094 | 0.121 | n/a |
| 0.6 | Y | 0.1 | *β_1_* | 0.468 | 0.468 | 0.061 | 0.042 | 0.042 | 0.005 | 0.089 | 0.089 | 0.083 | 0.054 | 0.055 | 0.052 |
|  |  | 0.1 | *β_2_* | -0.119 | -0.119 | -0.208 | -0.013 | -0.013 | -0.023 | 0.110 | 0.111 | 0.113 | 0.122 | 0.122 | 0.115 |
|  |  | 1.0 | *β_1_* | 4.498 | 4.489 | 0.920 | 0.394 | 0.394 | 0.073 | 0.088 | 0.088 | 0.079 | 0.054 | 0.054 | 0.049 |
|  |  | 1.0 | *β_2_* | -0.635 | -0.630 | -1.467 | -0.066 | -0.066 | -0.142 | 0.105 | 0.105 | 0.097 | 0.111 | 0.111 | 0.108 |
|  |  | 3.0 | *β_1_* | 9.547 | 9.501 | 1.835 | 0.779 | 0.779 | 0.178 | 0.082 | 0.082 | 0.097 | 0.055 | 0.055 | 0.047 |
|  |  | 3.0 | *β_2_* | -0.827 | -0.816 | -2.260 | -0.079 | -0.079 | -0.199 | 0.096 | 0.097 | 0.088 | 0.097 | 0.097 | 0.101 |
|  |  | 5.0 | *β_1_* | 11.691 | 11.668 | 2.069 | 0.932 | 0.932 | 0.229 | 0.080 | 0.080 | 0.111 | 0.056 | 0.057 | 0.046 |
|  |  | 5.0 | *β_2_* | -0.773 | -0.771 | -2.330 | -0.072 | -0.072 | -0.209 | 0.093 | 0.094 | 0.090 | 0.094 | 0.094 | 0.099 |
| 1.0 | C | 0.1 | *β_1_* | 0.531 | -0.028 | n/a | 0.074 | -0.003 | n/a | 0.138 | 0.121 | n/a | 0.075 | 0.065 | n/a |
|  |  | 0.1 | *β_2_* | -0.150 | 0.035 | n/a | -0.022 | 0.005 | n/a | 0.147 | 0.151 | n/a | 0.168 | 0.162 | n/a |
|  |  | 1.0 | *β_1_* | 4.802 | -0.114 | n/a | 0.654 | -0.014 | n/a | 0.136 | 0.119 | n/a | 0.072 | 0.062 | n/a |
|  |  | 1.0 | *β_2_* | -0.772 | 1.172 | n/a | -0.104 | 0.213 | n/a | 0.135 | 0.181 | n/a | 0.148 | 0.173 | n/a |
|  |  | 3.0 | *β_1_* | 10.930 | 0.149 | n/a | 1.299 | 0.017 | n/a | 0.119 | 0.111 | n/a | 0.067 | 0.059 | n/a |
|  |  | 3.0 | *β_2_* | -1.119 | 3.087 | n/a | -0.126 | 0.561 | n/a | 0.113 | 0.182 | n/a | 0.119 | 0.170 | n/a |
|  |  | 5.0 | *β_1_* | 13.439 | 0.484 | n/a | 1.549 | 0.053 | n/a | 0.115 | 0.109 | n/a | 0.066 | 0.060 | n/a |
|  |  | 5.0 | *β_2_* | -1.092 | 4.081 | n/a | -0.115 | 0.679 | n/a | 0.105 | 0.166 | n/a | 0.111 | 0.163 | n/a |
| 1.0 | Y | 0.1 | *β_1_* | 0.516 | 0.516 | 0.049 | 0.071 | 0.071 | 0.006 | 0.137 | 0.137 | 0.127 | 0.075 | 0.076 | 0.071 |
|  |  | 0.1 | *β_2_* | -0.149 | -0.145 | -0.214 | -0.021 | -0.021 | -0.032 | 0.144 | 0.145 | 0.150 | 0.168 | 0.169 | 0.154 |
|  |  | 1.0 | *β_1_* | 4.805 | 4.793 | 0.848 | 0.654 | 0.654 | 0.100 | 0.136 | 0.136 | 0.118 | 0.072 | 0.072 | 0.066 |
|  |  | 1.0 | *β_2_* | -0.786 | -0.784 | -1.449 | -0.106 | -0.106 | -0.180 | 0.135 | 0.136 | 0.124 | 0.148 | 0.148 | 0.142 |
|  |  | 3.0 | *β_1_* | 10.850 | 10.786 | 2.177 | 1.297 | 1.297 | 0.274 | 0.120 | 0.120 | 0.126 | 0.067 | 0.068 | 0.060 |
| 1.0 | Y | 3.0 | *β_2_* | -1.118 | -1.107 | -2.289 | -0.128 | -0.128 | -0.242 | 0.115 | 0.115 | 0.106 | 0.119 | 0.120 | 0.130 |
|  |  | 5.0 | *β_1_* | 13.248 | 13.205 | 2.613 | 1.550 | 1.550 | 0.368 | 0.117 | 0.117 | 0.141 | 0.066 | 0.067 | 0.057 |
|  |  | 5.0 | *β_2_* | -1.105 | -1.105 | -2.425 | -0.118 | -0.118 | -0.251 | 0.106 | 0.107 | 0.104 | 0.111 | 0.112 | 0.125 |

* PMM only applied in settings in which *Y* was partially observed.

Monte Carlo SE of bias is at most 0.006 for *β_1_* and *β_2._*

*Table S4. Simulation results for Scenario 2: Y, X, and C are continuous, Y depends on X, C, and C^2^, and X depends on C.*

*Estimates of standardised bias (bias/ SD(*${\hat{\text{β}}}_{\text{i}}$*)), bias of* ${\hat{\text{β}}}_{\text{i}}$*, SD(*${\hat{\text{β}}}_{\text{i}}$*), and model-based standard error (SE) are shown (i = 1 or 2), for complete records analysis (CRA), MI using draws from a linear imputation model (MI), and MI using type 1 predictive mean matching (PMM).*

*Results are shown for different strengths of the association (τ) between C and missingness of X or Y.*

*True values of the exposure and confounder coefficients for the fitted analysis model (E(Y) = β_0_ + β_1_ X + β_2_ C) are β_1_ = 0.4, β_2_ = 1.4.*

| **Partially observed**  **variable** | ***τ*** | **Par.** | **Standardised Bias** | | | **Bias of** ${\hat{\text{β}}}_{\text{i}}$ | | | **SD(**${\hat{\text{β}}}_{\text{i}}$**)** | | | **Model-based SE** | | |
| --- | --- | --- | --- | --- | --- | --- | --- | --- | --- | --- | --- | --- | --- | --- |
|  |  |  | **CRA** | **MI** | **PMM** | **CRA** | **MI** | **PMM** | **CRA** | **MI** | **PMM** | **CRA** | **MI** | **PMM** |
| X | 0.1 | *β_1_* | -0.024 | -0.019 | -0.509 | -0.001 | -0.001 | -0.028 | 0.052 | 0.051 | 0.055 | 0.050 | 0.049 | 0.050 |
|  | 0.1 | *β_2_* | 0.418 | 0.080 | 0.342 | 0.039 | 0.007 | 0.030 | 0.094 | 0.085 | 0.087 | 0.070 | 0.064 | 0.065 |
|  | 1.0 | *β_1_* | -0.039 | 0.697 | 0.292 | -0.002 | 0.036 | 0.017 | 0.046 | 0.051 | 0.058 | 0.047 | 0.049 | 0.052 |
|  | 1.0 | *β_2_* | 3.592 | 0.007 | -0.049 | 0.324 | 0.001 | -0.004 | 0.090 | 0.083 | 0.087 | 0.068 | 0.060 | 0.063 |
|  | 3.0 | *β_1_* | -0.023 | 1.990 | 1.708 | -0.001 | 0.104 | 0.101 | 0.042 | 0.052 | 0.059 | 0.043 | 0.050 | 0.052 |
|  | 3.0 | *β_2_* | 8.080 | -0.037 | -0.382 | 0.691 | -0.003 | -0.031 | 0.086 | 0.077 | 0.081 | 0.069 | 0.055 | 0.057 |
|  | 5.0 | *β_1_* | -0.050 | 2.395 | 2.116 | -0.002 | 0.121 | 0.119 | 0.040 | 0.051 | 0.056 | 0.041 | 0.049 | 0.051 |
|  | 5.0 | *β_2_* | 9.493 | 0.017 | -0.308 | 0.789 | 0.001 | -0.024 | 0.083 | 0.075 | 0.079 | 0.069 | 0.053 | 0.055 |
| Y | 0.1 | *β_1_* | 0.012 | 0.009 | -0.133 | 0.001 | 0.000 | -0.007 | 0.051 | 0.051 | 0.052 | 0.050 | 0.050 | 0.047 |
|  | 0.1 | *β_2_* | 0.392 | 0.392 | 0.095 | 0.037 | 0.037 | 0.009 | 0.093 | 0.094 | 0.090 | 0.070 | 0.071 | 0.067 |
|  | 1.0 | *β_1_* | -0.017 | -0.020 | -1.120 | -0.001 | -0.001 | -0.051 | 0.047 | 0.047 | 0.046 | 0.047 | 0.047 | 0.045 |
|  | 1.0 | *β_2_* | 3.571 | 3.567 | 1.079 | 0.324 | 0.324 | 0.090 | 0.091 | 0.091 | 0.083 | 0.068 | 0.069 | 0.064 |
|  | 3.0 | *β_1_* | -0.045 | -0.045 | -1.825 | -0.002 | -0.002 | -0.081 | 0.043 | 0.043 | 0.044 | 0.043 | 0.043 | 0.044 |
|  | 3.0 | *β_2_* | 7.937 | 7.882 | 1.694 | 0.691 | 0.692 | 0.165 | 0.087 | 0.088 | 0.097 | 0.069 | 0.070 | 0.064 |
|  | 5.0 | *β_1_* | -0.032 | -0.033 | -1.900 | -0.001 | -0.001 | -0.084 | 0.041 | 0.041 | 0.044 | 0.041 | 0.042 | 0.044 |
|  | 5.0 | *β_2_* | 9.104 | 9.042 | 1.618 | 0.787 | 0.786 | 0.178 | 0.086 | 0.087 | 0.110 | 0.069 | 0.069 | 0.063 |

Monte Carlo SE of bias is at most 0.003 for *β_1_* and *β_2._*

*Table S5. Simulation results for Scenario 3: Y, X, and C are continuous, Y depends on X and C, and X depends on C^2^.*

*Estimates of standardised bias (bias/ SD(*${\hat{\text{β}}}_{\text{i}}$*)), bias of* ${\hat{\text{β}}}_{\text{i}}$*, SD(*${\hat{\text{β}}}_{\text{i}}$*), and model-based standard error (SE) are shown (i = 1 or 2), for complete records analysis (CRA), MI using draws from a linear imputation model (MI), and MI using type 1 predictive mean matching (PMM).*

*Results are shown for different strengths of the association (τ) between C and missingness of X or Y.*

*True values of the exposure and confounder coefficients for the fitted analysis model (E(Y) = β_0_ + β_1_ X + β_2_ C) are β_1_ = 0.4, β_2_ = 0.8.*

| **Partially observed**  **variable** | ***τ*** | **Par.** | **Standardised Bias** | | | **Bias of** ${\hat{\text{β}}}_{\text{i}}$ | | | **SD(**${\hat{\text{β}}}_{\text{i}}$**)** | | | **Model-based SE** | | |
| --- | --- | --- | --- | --- | --- | --- | --- | --- | --- | --- | --- | --- | --- | --- |
|  |  |  | **CRA** | **MI** | **PMM** | **CRA** | **MI** | **PMM** | **CRA** | **MI** | **PMM** | **CRA** | **MI** | **PMM** |
| X | 0.1 | *β_1_* | 0.060 | 0.050 | 0.049 | 0.001 | 0.001 | 0.001 | 0.022 | 0.021 | 0.021 | 0.022 | 0.021 | 0.021 |
|  | 0.1 | *β_2_* | -0.053 | -0.411 | -0.314 | -0.002 | -0.019 | -0.014 | 0.046 | 0.045 | 0.045 | 0.045 | 0.040 | 0.040 |
|  | 1.0 | *β_1_* | 0.040 | 0.688 | 0.139 | 0.001 | 0.016 | 0.004 | 0.023 | 0.024 | 0.027 | 0.024 | 0.023 | 0.024 |
|  | 1.0 | *β_2_* | -0.044 | -3.191 | -1.786 | -0.002 | -0.175 | -0.099 | 0.055 | 0.055 | 0.056 | 0.055 | 0.046 | 0.046 |
|  | 3.0 | *β_1_* | 0.031 | 1.592 | 0.358 | 0.001 | 0.046 | 0.012 | 0.028 | 0.029 | 0.035 | 0.029 | 0.029 | 0.026 |
|  | 3.0 | *β_2_* | -0.012 | -6.069 | -2.204 | -0.001 | -0.446 | -0.180 | 0.078 | 0.073 | 0.082 | 0.078 | 0.064 | 0.051 |
|  | 5.0 | *β_1_* | 0.050 | 1.820 | 0.769 | 0.001 | 0.055 | 0.029 | 0.029 | 0.030 | 0.037 | 0.030 | 0.031 | 0.026 |
|  | 5.0 | *β_2_* | -0.038 | -6.972 | -2.532 | -0.003 | -0.528 | -0.236 | 0.083 | 0.076 | 0.093 | 0.085 | 0.070 | 0.053 |
| Y | 0.1 | *β_1_* | 0.046 | 0.049 | -0.113 | 0.001 | 0.001 | -0.002 | 0.021 | 0.021 | 0.022 | 0.022 | 0.022 | 0.022 |
|  | 0.1 | *β_2_* | -0.060 | -0.066 | -0.068 | -0.003 | -0.003 | -0.003 | 0.046 | 0.046 | 0.046 | 0.045 | 0.045 | 0.045 |
|  | 1.0 | *β_1_* | 0.063 | 0.056 | 0.035 | 0.001 | 0.001 | 0.001 | 0.023 | 0.023 | 0.024 | 0.024 | 0.024 | 0.024 |
|  | 1.0 | *β_2_* | -0.046 | -0.040 | -0.046 | -0.003 | -0.002 | -0.003 | 0.056 | 0.056 | 0.058 | 0.054 | 0.055 | 0.055 |
|  | 3.0 | *β_1_* | 0.066 | 0.056 | 0.040 | 0.002 | 0.002 | 0.001 | 0.028 | 0.029 | 0.029 | 0.029 | 0.029 | 0.029 |
|  | 3.0 | *β_2_* | -0.069 | -0.058 | -0.108 | -0.005 | -0.005 | -0.009 | 0.079 | 0.080 | 0.082 | 0.078 | 0.079 | 0.078 |
|  | 5.0 | *β_1_* | 0.067 | 0.066 | 0.023 | 0.002 | 0.002 | 0.001 | 0.029 | 0.030 | 0.030 | 0.030 | 0.030 | 0.030 |
|  | 5.0 | *β_2_* | -0.059 | -0.060 | -0.118 | -0.005 | -0.005 | -0.010 | 0.083 | 0.084 | 0.085 | 0.085 | 0.086 | 0.084 |

Monte Carlo SE of bias is at most 0.003 for *β_1_* and *β_2._*

*Table S6. Simulation results for Scenario 4: Y and C are binary, X is continuous, log-odds of Y depends on X^2^, and X depends on C.*

*Estimates of standardised bias (bias/ SD(*${\hat{\text{β}}}_{\text{i}}$*)), bias of* ${\hat{\text{β}}}_{\text{i}}$*, SD(*${\hat{\text{β}}}_{\text{i}}$*), and model-based standard error (SE) are shown (i = 1 or 2), for complete records analysis (CRA), and MI using draws from a logistic imputation model (MI).*

*Results are shown for different strengths of the association (τ) between X and missingness of C or Y.*

*True values of the exposure and confounder coefficients for the fitted analysis model (logit{P(Y=1)} = β_0_ + β_1_ X + β_2_ C) are β_1_ = 0.5, β_2_ = 0.8.*

| **Partially observed**  **variable** | ***τ*** | **Par.** | **Standardised Bias** | | **Bias of** ${\hat{\text{β}}}_{\text{i}}$ | | **SD(**${\hat{\text{β}}}_{\text{i}}$**)** | | **Model-based SE** | |
| --- | --- | --- | --- | --- | --- | --- | --- | --- | --- | --- |
|  |  |  | **CRA** | **MI** | **CRA** | **MI** | **CRA** | **MI** | **CRA** | **MI** |
| C | 0.1 | *β_1_* | 0.478 | 0.228 | 0.044 | 0.018 | 0.092 | 0.077 | 0.091 | 0.078 |
|  | 0.1 | *β_2_* | 0.140 | 0.159 | 0.025 | 0.028 | 0.178 | 0.178 | 0.187 | 0.188 |
|  | 1.0 | *β_1_* | 2.571 | 0.278 | 0.280 | 0.022 | 0.109 | 0.078 | 0.106 | 0.078 |
|  | 1.0 | *β_2_* | -0.030 | 0.129 | -0.005 | 0.024 | 0.185 | 0.184 | 0.188 | 0.189 |
|  | 3.0 | *β_1_* | 4.637 | 0.320 | 0.598 | 0.024 | 0.129 | 0.076 | 0.137 | 0.078 |
|  | 3.0 | *β_2_* | -0.024 | 0.198 | -0.004 | 0.037 | 0.185 | 0.186 | 0.189 | 0.188 |
|  | 5.0 | *β_1_* | 5.099 | 0.334 | 0.726 | 0.025 | 0.142 | 0.076 | 0.154 | 0.078 |
|  | 5.0 | *β_2_* | -0.024 | 0.210 | -0.004 | 0.039 | 0.186 | 0.185 | 0.191 | 0.190 |
| Y | 0.1 | *β_1_* | 0.466 | 0.468 | 0.043 | 0.043 | 0.092 | 0.092 | 0.091 | 0.092 |
|  | 0.1 | *β_2_* | 0.125 | 0.124 | 0.023 | 0.023 | 0.184 | 0.185 | 0.187 | 0.188 |
|  | 1.0 | *β_1_* | 2.720 | 2.715 | 0.283 | 0.283 | 0.104 | 0.104 | 0.107 | 0.108 |
|  | 1.0 | *β_2_* | 0.000 | -0.003 | 0.000 | -0.001 | 0.185 | 0.185 | 0.189 | 0.190 |
|  | 3.0 | *β_1_* | 4.583 | 4.550 | 0.599 | 0.597 | 0.131 | 0.131 | 0.136 | 0.138 |
|  | 3.0 | *β_2_* | -0.021 | -0.022 | -0.004 | -0.004 | 0.187 | 0.188 | 0.189 | 0.189 |
|  | 5.0 | *β_1_* | 5.041 | 5.004 | 0.728 | 0.726 | 0.144 | 0.145 | 0.154 | 0.155 |
|  | 5.0 | *β_2_* | -0.011 | -0.006 | -0.002 | -0.001 | 0.186 | 0.186 | 0.191 | 0.192 |

Monte Carlo SE of bias is at most 0.006 for *β_1_* and *β_2._*

*Table S7. Scenario 1: Sensitivity and type 1 error of various methods for examining analysis model mis-specification when fitting a linear regression model. Results are shown when either C or Y were partially observed, for different strengths of the association (τ) between X and missingness of C or Y, and for different strengths of the non-linear association (φ) between X and Y.*

| ***φ*** | **Partially observed variable** | ***τ*** | **Sensitivity (type 1 error)** | | | | | | | | | | | |
| --- | --- | --- | --- | --- | --- | --- | --- | --- | --- | --- | --- | --- | --- | --- |
|  |  |  |  | |  | |  | | **Model for regression of residuals on fitted values** | | | | | |
|  |  |  | 1. **Link** | | 1. **Shapiro-Wilk** | | 1. **Hetero-skedasticity** | | 1. **FP deg. 2 method** | | 1. **FP deg. 1 method** | | 1. **Grouped regression** | |
| 0.1 | *C* | 0.1 | 0.97 | (0.00) | 0.05 | (0.05) | 0.06 | (0.04) | 0.85 | (0.02) | 0.10 | (0.04) | 0.46 | (0.01) |
|  |  | 1 | 0.93 | (0.00) | 0.05 | (0.05) | 0.05 | (0.05) | 0.71 | (0.02) | 0.05 | (0.05) | 0.30 | (0.01) |
|  |  | 3 | 0.64 | (0.00) | 0.05 | (0.04) | 0.06 | (0.05) | 0.31 | (0.01) | 0.04 | (0.04) | 0.11 | (0.01) |
|  |  | 5 | 0.48 | (0.00) | 0.05 | (0.04) | 0.06 | (0.05) | 0.18 | (0.02) | 0.03 | (0.04) | 0.08 | (0.01) |
|  | *Y* | 0.1 | 0.97 | (0.00) | 0.06 | (0.06) | 0.08 | (0.05) | 0.86 | (0.02) | 0.10 | (0.04) | 0.45 | (0.01) |
|  |  | 1 | 0.92 | (0.00) | 0.04 | (0.05) | 0.05 | (0.04) | 0.71 | (0.01) | 0.09 | (0.04) | 0.33 | (0.02) |
|  |  | 3 | 0.66 | (0.00) | 0.05 | (0.04) | 0.05 | (0.06) | 0.33 | (0.01) | 0.04 | (0.02) | 0.13 | (0.01) |
|  |  | 5 | 0.49 | (0.00) | 0.04 | (0.04) | 0.05 | (0.05) | 0.16 | (0.01) | 0.03 | (0.03) | 0.07 | (0.02) |
| 0.6 | *C* | 0.1 | 1.00 | (0.02) | 1.00 | (0.05) | 0.36 | (0.05) | 1.00 | (0.04) | 1.00 | (0.05) | 1.00 | (0.02) |
|  |  | 1 | 1.00 | (0.01) | 1.00 | (0.04) | 0.34 | (0.05) | 1.00 | (0.04) | 1.00 | (0.04) | 1.00 | (0.01) |
|  |  | 3 | 1.00 | (0.00) | 0.92 | (0.05) | 0.62 | (0.04) | 1.00 | (0.03) | 1.00 | (0.03) | 1.00 | (0.01) |
|  |  | 5 | 1.00 | (0.00) | 0.70 | (0.04) | 0.78 | (0.04) | 1.00 | (0.02) | 1.00 | (0.04) | 1.00 | (0.01) |
|  | *Y* | 0.1 | 1.00 | (0.02) | 1.00 | (0.05) | 0.35 | (0.04) | 1.00 | (0.03) | 1.00 | (0.04) | 1.00 | (0.02) |
|  |  | 1 | 1.00 | (0.01) | 1.00 | (0.05) | 0.35 | (0.04) | 1.00 | (0.03) | 1.00 | (0.04) | 1.00 | (0.02) |
|  |  | 3 | 1.00 | (0.00) | 0.92 | (0.05) | 0.60 | (0.04) | 1.00 | (0.03) | 1.00 | (0.03) | 1.00 | (0.01) |
|  |  | 5 | 1.00 | (0.00) | 0.72 | (0.05) | 0.78 | (0.04) | 1.00 | (0.01) | 1.00 | (0.03) | 1.00 | (0.01) |
| 1.0 | *C* | 0.1 | 1.00 | (0.05) | 1.00 | (0.04) | 0.51 | (0.05) | 1.00 | (0.05) | 1.00 | (0.04) | 1.00 | (0.03) |
|  |  | 1 | 1.00 | (0.03) | 1.00 | (0.05) | 0.54 | (0.04) | 1.00 | (0.04) | 1.00 | (0.03) | 1.00 | (0.01) |
|  |  | 3 | 1.00 | (0.00) | 1.00 | (0.04) | 0.85 | (0.04) | 1.00 | (0.03) | 1.00 | (0.03) | 1.00 | (0.01) |
|  |  | 5 | 1.00 | (0.00) | 1.00 | (0.04) | 0.98 | (0.05) | 1.00 | (0.02) | 1.00 | (0.04) | 1.00 | (0.01) |
|  | *Y* | 0.1 | 1.00 | (0.03) | 1.00 | (0.04) | 0.53 | (0.05) | 1.00 | (0.04) | 1.00 | (0.04) | 1.00 | (0.01) |
|  |  | 1 | 1.00 | (0.03) | 1.00 | (0.04) | 0.51 | (0.05) | 1.00 | (0.03) | 1.00 | (0.04) | 1.00 | (0.02) |
|  |  | 3 | 1.00 | (0.01) | 1.00 | (0.05) | 0.85 | (0.04) | 1.00 | (0.04) | 1.00 | (0.04) | 1.00 | (0.01) |
|  |  | 5 | 1.00 | (0.00) | 1.00 | (0.04) | 0.97 | (0.05) | 1.00 | (0.03) | 1.00 | (0.04) | 1.00 | (0.01) |

*Table S8. Scenario 1: Sensitivity and type 1 error of various methods for examining imputation model mis-specification when fitting a logistic regression model, with C partially observed. Results are shown for different strengths of the association (τ) between X and missingness of C, and for different strengths of the non-linear association (φ) between X and Y.*

| ***φ*** | ***τ*** | **Sensitivity (type 1 error)** | | | | | |
| --- | --- | --- | --- | --- | --- | --- | --- |
|  |  | 1. **Link** | | 1. **Hinkley*** | | 1. **Hosmer-Lemeshow** | |
| 0.1 | 0.1 | 0.10 | (0.03) | 0.03 | (0.03) | 0.07 | (0.03) |
|  | 1 | 0.10 | (0.03) | 0.03 | (0.03) | 0.05 | (0.04) |
|  | 3 | 0.06 | (0.04) | 0.03 | (0.03) | 0.04 | (0.03) |
|  | 5 | 0.07 | (0.03) | 0.03 | (0.03) | 0.05 | (0.04) |
| 0.6 | 0.1 | 0.86 | (0.02) | 0.58 | (0.02) | 0.48 | (0.04) |
|  | 1 | 0.81 | (0.02) | 0.46 | (0.02) | 0.45 | (0.04) |
|  | 3 | 0.62 | (0.03) | 0.24 | (0.02) | 0.26 | (0.04) |
|  | 5 | 0.51 | (0.03) | 0.17 | (0.02) | 0.19 | (0.06) |
| 1.0 | 0.1 | 0.98 | (0.02) | 0.91 | (0.01) | 0.78 | (0.03) |
|  | 1 | 0.96 | (0.02) | 0.87 | (0.01) | 0.73 | (0.04) |
|  | 3 | 0.90 | (0.03) | 0.68 | (0.02) | 0.58 | (0.04) |
|  | 5 | 0.85 | (0.03) | 0.53 | (0.02) | 0.46 | (0.04) |

* Model used in the Hinkley method did not converge in 3% of simulations

*Table S9. Scenarios 2-4: Sensitivity and type 1 error of methods for examining analysis model mis-specification when Y (continuous in Scenarios 2 and 3, and binary in Scenario 4) is partially observed, and imputation model mis-specification when continuous X or binary C are partially observed (method for continuous variables: fitting a degree two fractional polynomial in the regression of the residuals on the fitted values; method for binary variables: Pregibon’s “link” method). Results are shown for different strengths of the missingness association (τ).*

*Note that, because Scenario 1 results for the analysis model specification were so similar when either Y or C were partially observed, in all other scenarios we only examined the specification of the analysis model when Y was partially observed.*

| **Scenario** | **Partially observed**  **variable** | ***τ*** | **Sensitivity (type 1 error)** | |
| --- | --- | --- | --- | --- |
| 2 | Y (continuous) | 0.1 | 1.00 | (0.01) |
|  |  | 1 | 1.00 | (0.01) |
|  |  | 3 | 1.00 | (0.01) |
|  |  | 5 | 1.00 | (0.01) |
|  | X (continuous) | 0.1 | 1.00 | (0.01) |
|  |  | 1 | 1.00 | (0.01) |
|  |  | 3 | 1.00 | (0.01) |
|  |  | 5 | 1.00 | (0.01) |
| 3 | Y* (continuous) | 0.1 | NA | (0.01) |
|  |  | 1 | NA | (0.02) |
|  |  | 3 | NA | (0.02) |
|  |  | 5 | NA | (0.02) |
|  | X (continuous) | 0.1 | 1.00 | (0.01) |
|  |  | 1 | 1.00 | (0.01) |
|  |  | 3 | 1.00 | (0.01) |
|  |  | 5 | 1.00 | (0.01) |
| 4 | Y (binary) | 0.1 | 1.00 | (0.05) |
|  |  | 1 | 1.00 | (0.04) |
|  |  | 3 | 0.80 | (0.02) |
|  |  | 5 | 0.54 | (0.02) |
|  | C (binary) | 0.1 | 0.07 | (0.00) |
|  |  | 1 | 0.04 | (0.00) |
|  |  | 3 | 0.03 | (0.00) |
|  |  | 5 | 0.03 | (0.00) |

* In scenario 3, the chosen analysis model correctly specifies the relationship between *Y, X* and *C*. Hence, sensitivity equals type 1 error in this case.

*
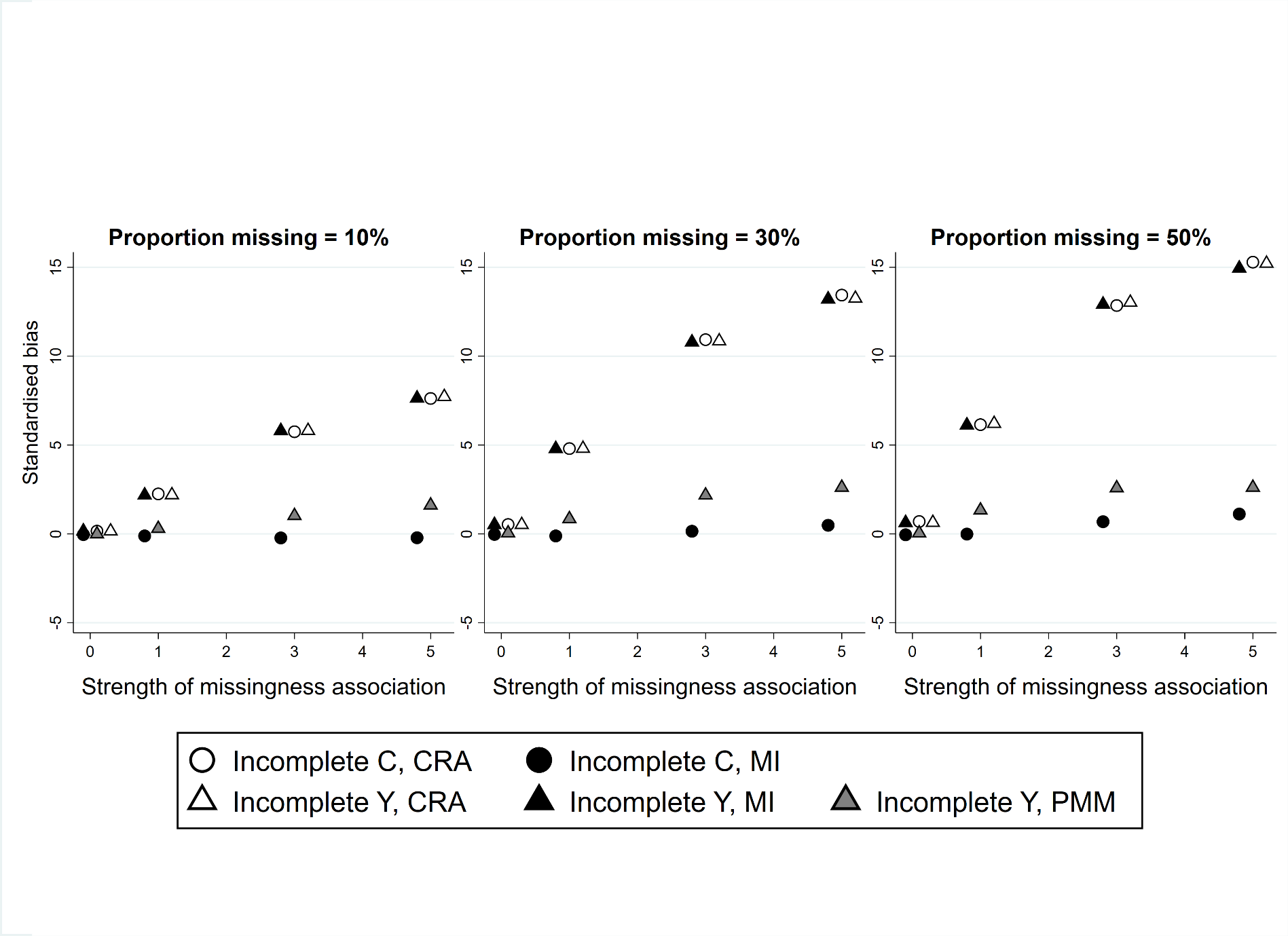
Figure S5. Standardised bias of complete records analysis (CRA), multiple imputation (MI), and predictive mean matching (PMM) estimates of parameter* $\text{β}_{\text{X}}$*, plotted against the strength of the missingness association, for different proportions of missing data, when the strength of the non-linear association (φ) between X and Y equals 1, given a quadratic relationship between continuous variables Y and X,* *and either C or Y partially observed.*

*Some overlapping points have been horizontally jittered.*

***Section S5. Stata code for the simulation study***

******Data generation for sim study**********

*Define postfile to store results

tempname simloop

postfile `simloop' float(sampno c3bin c3cts x1_b x1_c x1_d y1_b1_p1 y1_b1_p6 y1_b1_1 y1_c1 y1_d1 y1_b1_bin) using "sim1_DGM1000.dta", replace

*Also store rngstate

tempname simseed

postfile `simseed' str2000(s1 s2 s3) using "sim1_DGM1000_seedfile.dta", replace

*Create a temporary file for storing results for each iteration

tempfile tmpDGM

forvalues i=1/1000 {

*record seed at start of each iteration

post `simseed' (substr(c(rngstate),1,2000)) (substr(c(rngstate),2001,2000)) (substr(c(rngstate),4001,.))

clear

quietly set obs 1000

*Sample no

gen sampno=`i'

gen c3bin=rbinomial(1,0.5)

gen c3cts=rnormal(0.5,1)

*Specify DGM for X: either (i) X = C + ε (C either binary or cts) or X = C^2 + ε, where ε ~ N(0,1)

gen x1_b=rnormal(c3bin,1)

gen x1_c=rnormal(c3cts,1)

gen x1_d=rnormal(c3cts^2,1)

****** Scenario 1 - Y ~ N(-0.4 + 0.4 X + 0.8 C + phi X^2,1)

gen y1_b1_p1=rnormal(-0.4 + 0.4*x1_b + 0.8*c3bin + 0.1*x1_b^2,1)

gen y1_b1_p6=rnormal(-0.4 + 0.4*x1_b + 0.8*c3bin + 0.6*x1_b^2,1)

gen y1_b1_1=rnormal(-0.4 + 0.4*x1_b + 0.8*c3bin + 1.0*x1_b^2,1)

****** Scenario 2 - Y ~ N(-0.4 + 0.4 X + 0.8 C + 0.6 C^2,1)

gen y1_c1=rnormal(-0.4 + 0.4*x1_c + 0.8*c3cts + 0.6*c3cts^2,1)

****** Scenario 3 - Y ~ N(-0.4 + 0.4 X + 0.8 C,1) ******

gen y1_d1=rnormal(-0.4 + 0.4*x1_d + 0.8*c3cts,1)

****** Scenario 4 - Y ~logit-1(-0.4 + 0.4 X + 0.6 X^2 + 0.8 C)

gen y1_b1_bin=rbinomial(1,invlogit(-0.4 + 0.4*x1_b + 0.8*c3bin + 0.5*x1_b^2))

quietly save `tmpDGM', replace

use sim1_DGM1000, clear

append using `tmpDGM'

quietly save sim1_DGM1000, replace

}

postclose `simseed'

postclose `simloop'

********Run simulation study********

*To avoid repetition, code is supplied for Scenario 1 only

*Code is easily adaptable for Scenarios 2-4

*Define postfile to store results

tempname simloop

postfile `simloop' int(i) float(tau phi) str7(correct dgm_y dgm_x missvar missmethod) ///

float(const se_const beta_x se_x beta_c3 se_c3 beta_xsq se_xsq link swilk het FP_d2 FP_d1 quint link_lgt hinkley HL) using "sim1_yctsB1.dta", replace

*Also store rngstate

tempname simseed

postfile `simseed' str2000(s1 s2 s3) using "sim1_yctsB1_seedfile.dta", replace

*Create a temporary file for storing imputed data

tempfile tmpfull

forvalues i=1/1000 {

di "`i'"

*record seed at start of each iteration

post `simseed' (substr(c(rngstate),1,2000)) (substr(c(rngstate),2001,2000)) (substr(c(rngstate),4001,.))

foreach phi of numlist 1.0 0.6 0.1 {

foreach tau of numlist 5 3 1 0.1 {

*********SCENARIO 1. ******************

* First using correctly specified models to calculate type 1 error

* Code shown only for settings in which C is partially observed

* Code is very similar when Y is partially observed

quietly use sim1_DGM1000 if sampno==`i', clear

quietly gen y=y1_b1_p1 if `phi’ < 0.2

quietly replace y=y1_b1_p6 if `phi’ > 0.2 & `phi’ < 0.7

quietly replace y=y1_b1_1 if `phi’ > 0.7

gen x=x1_b

gen xsq=x1_b^2

if `tau' < 0.2 local alpha = 8

else if `tau' > 0.2 & `tau' < 2 local alpha = 0.6

else if `tau' > 2 & `tau' < 4 local alpha = 0.2

else local alpha = 0.1

quietly gen r_c3=rbinomial(1,invlogit(`tau'*(`alpha' + x)))

* Allowable range for p is 1e-8 to 1-1e-8, so extreme values of x give missing values for r_c3

quietly replace r_c3=0 if invlogit(`tau'*(`alpha' + x)) < 0.00000001

gen c3miss=c3bin

quietly replace c3miss=. if r_c3==0

*Methods for examining model mis-specification

*Methods for analysis model mis-specification (linear regression)

quietly regress y x c3miss xsq

*store values for -post-

est store vals

*Link

quietly linktest

local link=(2 * ttail(r(df), abs(r(t))))

* Shapiro-Wilks

quietly swilk r

local swilk=r(p)

*Heteroskedasticity

quietly estat hettest

local het=r(p)

* FP methods

*first using default of dim(2)

quietly predict r,resid

quietly predict fit,xb

quietly fp <fit>, scale: regress r <fit>

local fp_d2=e(fp_compare)[1,4]

*then using dim(1)

drop fit_1 fit_2

quietly fp <fit>, dim(1) scale: regress r <fit>

local fp_d1=e(fp_compare)[1,4]

*Grouped residual method

xtile fit_q = fit, nq(5)

quietly anova r fit_q

local quint=Ftail(e(df_m),e(df_r),e(F))

*Methods for imputation model mis-specification (logistic regression)

quietly logistic c3miss y x xsq

*Link

quietly linktest

local link_lgt=(2 * (1-normal(abs(r(t)))))

*Hinkley – using capture as does not always converge

drop fit fit_1 fit_q

quietly predict fit,xb

capture {

quietly fp <fit>, scale: logit c3miss <fit> y x xsq

*Compare to model without fit

local hinkley=e(fp_compare)[1,4]

}

*If error, run rest of loop

if _rc!=0 {

local hinkley=.

}

*Hosmer-Lemeshow

quietly estat gof, group(10)

local HL=r(p)

*Restore regression estimates (for info) and post all ests

quietly est restore vals

post `simloop' (`i') (`tau') (`phi') ("Yes") ("B1") ("X1") ("C") ("CRA") ///

(_b[_cons]) (_se[_cons]) (_b[x]) (_se[x]) (_b[c3miss]) (_se[c3miss]) ///

(_b[xsq]) (_se[xsq]) (`link') (`swilk') (`het') (`fp_d2') (`fp_d1') (`quint') (`link_lgt') (`hinkley') (`HL')

*****USING INCORRECT MODELS****************

*1. Missingness in outcome Y

drop _all

quietly use sim1_DGM1000 if sampno==`i', clear

gen x=x1_b

if `tau' < 0.2 local alpha = 8

else if `tau' > 0.2 & `tau’ < 2 local alpha = 0.6

else if `tau' > 2 & `tau’ < 4 local alpha = 0.2

else local alpha = 0.1

quietly gen r_y=rbinomial(1,invlogit(`tau'*(`alpha' + x)))

* Allowable range for p is 1e-8 to 1-1e-8, so extreme values of x give missing values for r_y

quietly replace r_y=0 if invlogit(`tau'*(`alpha' + x)) < 0.00000001

quietly gen ymiss=y1_b1_p1 if `phi' < 0.2

quietly replace ymiss=y1_b1_p6 if `phi' > 0.2 & `phi' < 0.7

quietly replace ymiss=y1_b1_1 if `phi' > 0.7

quietly replace ymiss=. if r_y==0

*save for use with PMM later

quietly save `tmpfull', replace

*CRA

quietly regress ymiss x c3bin

*store values for -post-

est store vals

*Methods for analysis model mis-specification (linear regression)

*Link

quietly linktest

local link=(2 * ttail(r(df), abs(r(t))))

* Shapiro-Wilks

quietly swilk r

local swilk=r(p)

*Heteroskedasticity

quietly estat hettest

local het=r(p)

* FP methods

quietly predict r,resid

quietly predict fit,xb

*first using default of dim(2)

quietly fp <fit>, scale: regress r <fit>

local fp_d2=e(fp_compare)[1,4]

*then using dim(1)

drop fit_1 fit_2

quietly fp <fit>, dim(1) scale: regress r <fit>

local fp_d1=e(fp_compare)[1,4]

*Grouped residual method

xtile fit_q = fit, nq(5)

quietly anova r fit_q

local quint=Ftail(e(df_m),e(df_r),e(F))

*Restore regression estimates and post all ests

quietly est restore vals

post `simloop' (`i') (`tau') (`phi') ("No") ("B1") ("X1") ("Y") ("CRA") ///

(_b[_cons]) (_se[_cons]) (_b[x]) (_se[x]) (_b[c3bin]) (_se[c3bin]) ///

(0) (0) (`link') (`swilk') (`het') (`fp_d2') (`fp_d1') (`quint') (0) (0) (0)

*** MI linear imputation model ***

quietly mi set flong

quietly mi register imputed ymiss

quietly mi register regular x c3bin

quietly mi impute chained (regress) ymiss = x c3bin, add(30)

quietly mi estimate: regress ymiss x c3bin

post `simloop' (`i') (`tau') (`phi') ("No") ("B1") ("X1") ("Y") ("MI") ///

(e(b_mi)[1,3]) (sqrt(e(V_mi)[3,3])) (e(b_mi)[1,1]) (sqrt(e(V_mi)[1,1])) ///

(e(b_mi)[1,2]) (sqrt(e(V_mi)[2,2])) ///

(0) (0) (0) (0) (0) (0) (0) (0) (0) (0) (0)

*** PMM ****

*Note, type 1 PMM can only be run in ice not mi impute

*restore simulated data i.e. before imputation

use `tmpfull', clear

quietly ice ymiss x c3bin, saving(`tmpfull', replace) m(30) match matchpool(5) uvisopts(matchtype(1))

use `tmpfull', clear

quietly mi import ice, automatic

quietly mi estimate: regress ymiss x c3bin

post `simloop' (`i') (`tau') (`phi') ("No") ("B1") ("X1") ("Y") ("PMM") ///

(e(b_mi)[1,3]) (sqrt(e(V_mi)[3,3])) (e(b_mi)[1,1]) (sqrt(e(V_mi)[1,1])) ///

(e(b_mi)[1,2]) (sqrt(e(V_mi)[2,2])) ///

(0) (0) (0) (0) (0) (0) (0) (0) (0) (0) (0)

*2. Missingness in confounder C

drop _all

quietly use sim1_DGM1000 if sampno==`i', clear

quietly gen y=y1_b1_p1 if `phi' < 0.2

quietly replace y=y1_b1_p6 if `phi' > 0.2 & `phi' < 0.7

quietly replace y=y1_b1_1 if `phi' > 0.7

gen x=x1_b

if `tau' < 0.2 local alpha = 8

else if `tau' > 0.2 & `tau' < 2 local alpha = 0.6

else if `tau' > 2 & `tau' < 4 local alpha = 0.2

else local alpha = 0.1

quietly gen r_c3=rbinomial(1,invlogit(`tau'*(`alpha' + x)))

* Allowable range for p is 1e-8 to 1-1e-8, so extreme values of x give missing values for r_c3

quietly replace r_c3=0 if invlogit(`tau'*(`alpha' + x)) < 0.00000001

gen c3miss=c3bin

quietly replace c3miss=. if r_c3==0

*CRA

quietly regress y x c3miss

*store values for -post-

est store vals

*Methods for analysis model mis-specification (linear regression)

quietly regress y x c3miss

*store values for -post-

est store vals

*Link

quietly linktest

local link=(2 * ttail(r(df), abs(r(t))))

* Shapiro-Wilks

quietly swilk r

local swilk=r(p)

*Heteroskedasticity

quietly estat hettest

local het=r(p)

* FP methods

*first using default of dim(2)

quietly predict r,resid

quietly predict fit,xb

quietly fp <fit>, scale: regress r <fit>

local fp_d2=e(fp_compare)[1,4]

*then using dim(1)

drop fit_1 fit_2

quietly fp <fit>, dim(1) scale: regress r <fit>

local fp_d1=e(fp_compare)[1,4]

*Grouped residual method

xtile fit_q = fit, nq(5)

quietly anova r fit_q

local quint=Ftail(e(df_m),e(df_r),e(F))

*Methods for imputation model mis-specification (logistic regression)

quietly logistic c3miss y x

*Link

quietly linktest

local link_lgt=(2 * (1-normal(abs(r(t)))))

*Hinkley – using capture as does not always converge

drop fit fit_1 fit_q

quietly predict fit,xb

capture {

quietly fp <fit>, scale: logit c3miss <fit> y x

*Compare to model without fit

local hinkley=e(fp_compare)[1,4]

}

*If error, run rest of loop

if _rc!=0 {

local hinkley=.

}

*Hosmer-Lemeshow

quietly estat gof, group(10)

local HL=r(p)

*Restore regression estimates and post all ests

quietly est restore vals

post `simloop' (`i') (`tau') (`phi') ("No") ("B1") ("X1") ("C") ("CRA") ///

(_b[_cons]) (_se[_cons]) (_b[x]) (_se[x]) (_b[c3miss]) (_se[c3miss]) ///

(0) (0) (`link') (`swilk') (`het') (`fp_d2') (`fp_d1') (`quint') (`link_lgt’) (`hinkley’) (`HL’)

*MI linear imputation model

*Note PMM not used because C is binary in scenario 1

quietly mi set flong

quietly mi register imputed c3miss

quietly mi register regular y x

quietly mi impute chained (logit) c3miss = y x, add(30)

quietly mi estimate: regress y x c3miss

post `simloop' (`i') (`tau') (`phi') ("No") ("B1") ("X1") ("C") ("MI") ///

(e(b_mi)[1,3]) (sqrt(e(V_mi)[3,3])) (e(b_mi)[1,1]) (sqrt(e(V_mi)[1,1])) ///

(e(b_mi)[1,2]) (sqrt(e(V_mi)[2,2])) ///

(0) (0) (0) (0) (0) (0) (0) (0) (0) (0) (0)

}

}

}

postclose `simloop'

postclose `simseed'

***Section S6. Stata code for the real data analysis***

*Acupuncture data can be accessed here: *https://www.ncbi.nlm.nih.gov/pmc/articles/PMC1489946/#S1

use acupuncture, clear

*CRA

regress pk5 age sex migraine chronicity pk1 group

* Examining analysis/imputation model specification

quietly predict r,resid

quietly predict fit,xb

fp <fit>, scale: regress r <fit>

*MI linear imputation model

quietly mi set flong

quietly mi register imputed pk5

quietly mi register regular age sex migraine chronicity pk1 group

mi impute chained (regress) pk5=age sex migraine chronicity pk1 group, add(25)

mi estimate: regress pk5 age sex migraine chronicity pk1 group

*PMM

*Note, type 1 PMM can only be run in ice not mi impute

*First restore data i.e. before imputation

use acupuncture, clear

ice pk5 age sex migraine chronicity pk1 group, saving(tmp, replace) m(25) match matchpool(5) uvisopts(matchtype(1))

use tmp, clear

quietly mi import ice, automatic

mi estimate: regress pk5 age sex migraine chronicity pk1 group

*Check functional form of each continuous variable

use acupuncture, clear

fp <age>, scale: regress pk5 <age> sex migraine chronicity pk1 group

*No evidence against linear form

fp <chronicity>, scale: regress pk5 age sex migraine <chronicity> pk1 group

*No evidence against linear form

fp <pk1>: regress pk5 age sex migraine chronicity <pk1> group

*Suggests using squared version of pk1

gen pk1_sq=pk1^2

*Examine specification of this model

regress pk5 age sex migraine chronicity pk1 pk1_sq group

drop r fit

quietly predict r,resid

quietly predict fit,xb

fp <fit>, scale: regress r <fit>

/* Finally, add squared term to the imputation model*/

quietly mi set flong

quietly mi register imputed pk5

quietly mi register regular age sex migraine chronicity pk1 group pk1_sq

mi impute chained (regress) pk5=age sex migraine chronicity pk1 group pk1_sq, add(25)

mi estimate: regress pk5 age sex migraine chronicity pk1 group
